# Thermally Drawn Polymeric Catheters for MR‐Guided Cardiovascular Intervention

**DOI:** 10.1002/advs.202407704

**Published:** 2024-10-15

**Authors:** Mohamed E. M. K. Abdelaziz, Libaihe Tian, Thomas Lottner, Simon Reiss, Timo Heidt, Alexander Maier, Klaus Düring, Constantin von zur Mühlen, Michael Bock, Eric Yeatman, Guang‐Zhong Yang, Burak Temelkuran

**Affiliations:** ^1^ The Hamlyn Centre for Robotic Surgery Imperial College London London SW7 2AZ UK; ^2^ Department of Electrical and Electronic Engineering Faculty of Engineering Imperial College London London SW7 2AZ UK; ^3^ Department of Metabolism Digestion, and Reproduction Faculty of Medicine Imperial College London London SW7 2AZ UK; ^4^ Department of Diagnostic and Interventional Radiology Medical Physics Faculty of Medicine, University of Freiburg 79106 Freiburg Germany; ^5^ Department of Cardiology and Angiology University Heart Center Freiburg – Bad Krozingen Faculty of Medicine University of Freiburg 79106 Freiburg Germany; ^6^ MaRVis Interventional GmbH 82467 Garmisch‐Partenkirchen Germany; ^7^ Institute of Medical Robots Shanghai Jiao Tong University Shanghai 200240 China; ^8^ The Rosalind Franklin Institute Didcot OX11 0QS UK

**Keywords:** cardiovascular interventions, catheter, endovascular interventions, MRI, thermal drawings

## Abstract

Cardiovascular diseases (CVDs), including congenital heart diseases (CHD), present significant global health challenges, emphasizing the need for safe and effective treatment modalities. Fluoroscopy‐guided endovascular interventions are widely utilized but raise concerns about ionizing radiation, especially in pediatric cases. Magnetic resonance imaging (MRI) offers a radiation‐free alternative with superior soft tissue visualization and functional insights. However, the lack of compatible instruments remains a major obstacle. An adapted thermal drawing platform that enables low‐cost and rapid prototyping of instruments for MR‐guided endovascular interventions is introduced. This platform is demonstrated through the development of two exemplary catheter systems: a tendon‐driven steerable catheter with helical lumina and an active tracking Tiger‐shaped catheter with an embedded coaxial wire. These catheters exhibit mechanical properties comparable to commercial counterparts and show promising outcomes in both in vitro and in vivo feasibility testing. This scalable thermal drawing platform addresses the limitations of existing manufacturing approaches and facilitates the exploration of diverse designs, potentially accelerating advancements in catheter technologies for MR‐guided cardiovascular interventions.

## Graphical abstract

This research presents an adapted thermal drawing platform for cost‐effective and rapid prototyping of catheters for MR‐guided endovascular interventions. The benefits of the proposed platform are demonstrated in the development of advanced catheter systems, exhibiting excellent mechanical properties and MR visibility. Successful in vitro and in vivo testing underscores their potential for advanced cardiovascular procedures under MR guidance.

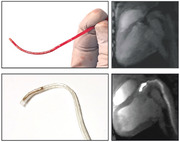

## 1 Introduction

Cardiovascular diseases (CVDs) account for ≈17.9 million deaths annually, standing as the primary global cause of mortality.^[^ ^1^ ^]^ Among these, congenital heart disease (CHD) is particularly notable, affecting 0.8% to 1.2% of live births globally, making it one of the most common congenital disorders.^[^ ^2^, ^3^ ^]^ Fluoroscopy‐guided endovascular interventions, known for their minimally invasive nature and effectiveness in reducing hospital stays, are widely utilized in treating CVDs. However, managing complex CHD often requires multiple palliative or corrective procedures throughout a patient's lifetime, necessitating repeated fluoroscopic catheterizations for both diagnostic and interventional purposes.

Endovascular fluoroscopy employs X‐ray imaging in conjunction with iodinated contrast agents. While these interventions are considered the “gold standard” in endovascular procedures,^[^ ^4^ ^]^ the ionizing radiation they involve poses a significant cancer risk to both patients and medical professionals.^[^ ^5^ ^]^ These concerns are especially critical in pediatric cases.^[^ ^6^ ^]^ Additionally, fluoroscopically guided cardiac catheterization, hindered by poor soft‐tissue contrast, presents challenges in the precise positioning of interventional tools. As a result, operators frequently rely on previously acquired contrast angiographic images or mental reconstruction of anatomical structures due to these constraints. Such limited visualization can prolong procedures, increase X‐ray exposure, and elevate the risk of complications. Furthermore, the use of radiopaque contrast agents for indirect vessel visualization can potentially impair kidney function.^[^ ^7^ ^]^

Magnetic resonance imaging (MRI) has attracted attention as a radiation‐free alternative to address these issues.^[^ ^8^ ^]^ MRI offers distinct advantages, including its unparalleled soft tissue visualization,^[^ ^9^, ^10^ ^]^ and ability to provide functional information such as blood flow, tissue oxygenation, diffusion, perfusion, and mechanical properties (elastography).^[^ ^11^, ^12^ ^]^ While gadolinium‐based contrast agents, commonly used in MR‐guided endovascular interventions, generally have a safer profile compared to iodinated contrast agents used in X‐ray imaging, they may still cause severe side effects in patients with impaired renal function.^[^ ^13^, ^14^ ^]^ However, recent studies have shown that MRIs can perform angiography and endovascular interventions with minimal or no contrast agent.^[^ ^14^ ^]^ The development of novel imaging sequences, with up to 20 images per second,^[^ ^15^, ^16^ ^]^ has further paved the way for real‐time MR‐guided interventions. Early feasibility studies have explored applications such as right heart catheterization,^[^ ^17^ ^]^ electrophysiology ablations,^[^ ^18^, ^19^ ^]^ endomyocardial biopsy,^[^ ^20^ ^]^ and coronary interventions.^[^ ^21^ ^]^

Despite its promising potential, MR‐guided interventions are hindered by the lack of key instruments that are compatible with MRI, such as guidewires, sheaths, and catheters.^[^ ^11^, ^22^ ^]^ Most commercially available instruments are designed for X‐ray‐guided procedures and rely on metallic materials, which often cause image artifacts that obscure important anatomical details. Additionally, elongated metallic structures such as guidewires can also lead to potentially dangerous radiofrequency‐induced heating in an MRI environment,^[^ ^23^ ^]^ especially when their length is close to the resonance length of the radio‐frequency fields.^[^ ^24^ ^]^ While polymers are considered MR‐safe alternatives to metals, directly replacing them comes with challenges related to MR visibility, mechanical performance, and manufacturing. Despite significant efforts to develop instruments compliant with MR‐Safe or MR‐Conditional standards according to ASTM F2503‐23,^[^ ^25^ ^]^ and to enhance instrument tracking, various challenges continue to limit the commercialization of these devices and the broader adoption of MR‐guided interventions.^[^ ^26^ ^]^

The development of fabrication approaches that allow fast and affordable prototyping is essential for both enabling and accelerating research surrounding these devices. This paper introduces an adapted, highly customized prototyping method for catheter fabrication employing fiber drawing technology—a well‐established and scalable technique. The thermal fiber drawing process has emerged as a pivotal tool for creating high‐aspect‐ratio devices with multi‐material and multifunctional capabilities across various applications.^[^ ^27^, ^28^, ^29^, ^30^ ^]^ The process starts with a macroscopic preform made of single or multiple materials, typically several centimeters in diameter and tens of centimeters in length, shaped to replicate the desired cross–sectional geometry of the final fiber. Through thermal softening and drawing in a viscous state, the preform attains the desired diameter and length while preserving the transverse geometry throughout the fiber.

Although high‐aspect‐ratio devices are common in the medical field, the application of thermal drawing for fabricating surgical instruments has been explored to a limited extent, since its first proposal in 2002.^[^ ^31^ ^]^ The use of thermally drawn catheters for endovascular interventions was initially described in.^[^ ^32^, ^33^ ^]^ Additionally, there have been advances in integrating multiple functions into a miniaturized thermally drawn robotic fiber,^[^ ^34^ ^]^ with one study demonstrating their feasibility for endovascular interventions in a phantom model.^[^ ^35^ ^]^ Other applications include a phantom study on auripuncture and drug delivery using a fiber‐based steerable robot,^[^ ^36^ ^]^ as well as the creation of a programmable bevel‐tip needle for neurosurgery through thermal drawing technology.^[^ ^37^ ^]^ These developments not only showcase the significant advantages of thermal drawing in miniaturizing complex structures but also highlight its great potential in medical device manufacturing.

This paper presents an adapted thermal drawing platform that enables cost‐effective and rapid prototyping of catheters for MR‐guided endovascular interventions. This platform is demonstrated through the development of two advanced catheter systems: a tendon‐driven steerable catheter with helical lumina and a Tiger‐shaped catheter with active tracking. The tendon‐driven catheter features a steerable tip, addressing the limitations associated with passive catheters with fixed tip shapes and the helical lumina design enhances the catheter's steering performance. The Tiger‐shaped catheter provides a compact integration of coaxial wire for active tracking within MR environments. The thermal drawing platform enables the creation of these challenging designs, including helical lumina and embedded coaxial wire, significantly simplifying the fabrication process compared to conventional methods. Both catheter systems, designed specifically for navigation, were successfully tested in vitro with a phantom and in vivo with a porcine model. The entire fabrication process was completed within a few weeks, demonstrating a low‐cost method from concept to prototype.

## 2 Results

### 2.1 Fabrication of Multi‐Lumen Tubing Using Thermal Drawing Technique

**Figure 1:**
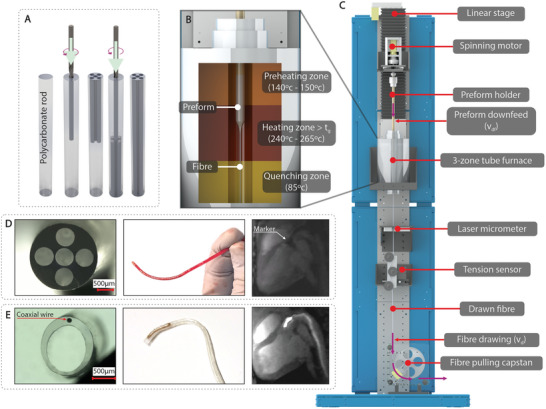
A) Photograph of the draw tower used for fabricating the fibers. B) Cross–sectional inset of the three‐zone tube furnace, indicating the corresponding preheating, heating, and quenching temperatures. C) CAD representation of the fiber draw tower. D) Steerable catheter: (left) microscopic view of the cross–sectional structure; (middle) photograph of the catheter tips showcasing their unique designs; (right) in vivo MR image of the catheters during testing in a living subject's heart. E) Active tracking catheter: (left) microscopic view of the cross–sectional structure; (middle) photograph of the catheter tips showcasing their unique designs; (right) in vivo MR image of the catheters during testing in a living subject's heart.

Utilizing the thermal drawing platform, two catheter systems were developed: a tendon‐driven steerable catheter and a Tiger‐shaped catheter with active tracking (**Figure** **1**D,E). The tendon‐driven catheter allows for multi‐directional steering of the tip, while the active tracking catheter allows MR trackability for a universal catheter tip design commonly referred to as “Tiger”.^[^ ^38^ ^]^ These catheters are constructed around a core component of multi‐lumen tubing, fabricated through our proposed thermal drawing technique. Unlike conventional extrusion, which is limited by challenges such as extrudate swelling and complex mold design,^[^ ^39^ ^]^ thermal drawing proved to be highly effective and cost‐efficient for producing high‐aspect‐ratio devices. In addition, we introduced two features to further enhance catcher functionality: a preform twist for the steerable catheter and wire feeding for the Tiger‐shaped catheter, which will be elaborated upon in the following sections.

The thermal drawing process was conducted, and a custom‐built draw tower equipped with a three‐zone tube furnace (detailed in Note S2 in Supporting Information) was employed (Figure 1C). The preforms were carefully fed into the furnace and subjected to controlled temperature profiles, including preheating, heating above the glass transition temperature, and quenching (Figure 1B). Real‐time monitoring of the drawn fibers’ outer diameter and tension using a laser micrometer, and a three‐wheel tension sensor enabled precise adjustments to the draw parameters, ensuring the production of high‐quality fibers.

For the steerable catheters, polycarbonate (PC) and polyetherimide (PEI) were selected due to their excellent mechanical properties and biocompatibility. The fabrication process began with the design and production of macroscopic preforms specifically tailored to accommodate the desired components. For the steerable catheters, the preforms were crafted with five precisely positioned (Figure S1A, Supporting Information) through holes, created by drilling (Figure 1A). The resulting catheter shaft (Figure 1D) was drawn from the preform, scaling down in diameter from 20 mm to ≈2 mm. Throughout the fabrication process, the cross–sectional geometry of the preform was preserved, with the diameter of the drilled holes reducing ≈10‐fold, from 6 mm to ≈0.6 mm, forming uniformly elongated channels.

For the Tiger‐shaped catheter, cyclic olefin copolymer elastomer (COCe) was chosen for the tip, with PC used for the shaft. The design of this catheter shaft featured a cross–sectional configuration with a narrow lumen for wire insertion and a wider lumen to allow the smooth passage of additional instruments. This functionality was achieved by drilling holes into off‐the‐shelf PC polymer rods, which were then thermally drawn into fibers of specified diameters. After drawing, the resulting catheter shaft (Figure 1E) had a diameter of 2.5 mm and contained two circular channels—one with a diameter of 0.2 mm, securely holding the wire, and another with a diameter of 1.9 mm to accommodate the passage of other instruments.

### 2.2 Multi‐Lumen Tubing with Helical Lumina Structure

Flexible tendon‐driven instruments often exhibit undesired motion, characterized by three phenomena: passive tip deflection, muscling, and curve alignment (Figure S4, Supporting Information). In the context of a catheter with a steerable tip, passive tip deflection results in uncontrollable movements of the catheter tip during navigation through tortuous pathways. Muscling refers to the unwanted lateral motion of both the catheter shaft and its distal end due to pull‐wire tensioning. Curve alignment describes the unintended rotational motion of the catheter shaft when the pull‐wire is tensioned to articulate the distal end in a specific direction. These issues significantly affect the catheter's tip steering performance but have often been overlooked in the literature due to manufacturing limitations. To address these challenges, Bogusky et al.^[^ ^40^ ^]^ proposed incorporating helically routed side lumina (pull‐wires) along the catheter shaft to distribute compressive loads and balance bending moments. Their approach involved a multi‐step fabrication method using main and supplemental mandrels fed through a rotating nose cone in a helical pattern.

**Figure 2:**
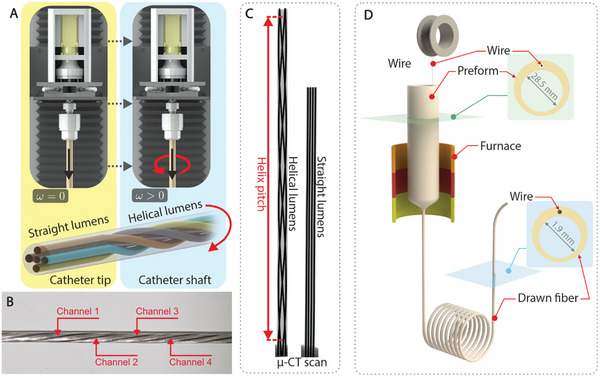
Preform twist and wire feeding in thermal drawing. A) Schematics illustrating the thermal drawing of multi‐lumen tubing with helical lumina structure: Parallel lumina of the distal end fabricated by switching off the spinning motor (*ω* = 0), while helical lumina of the catheter shaft fabricated by switching on the spinning motor (*ω* > 0). B) Photograph of multi‐lumen tubing with helical channels, visualizing using nitinol wires. C) (left) µ‐CT scan of multi‐lumen tubing with parallel channels embedded with four 0.4 mm nitinol wires for lumina visualization. (right) µ‐CT scan of multi‐lumen tubing with helical channels and a pitch size of 50 mm. D) CAD representation of the thermal drawing of multi‐lumen tubing with wire feeding and optical image of the resultant fibers.

Inspired by Bogusky et al.’s work, we propose a novel approach for fabricating multi‐lumen tubing with helically routed channels in a single step, aimed at suppressing the undesired motion caused by pull‐wire tension in our steerable catheter. This method utilizes the thermal drawing process, wherein the preform is spun during the drawing to create axially asymmetric fibers. Previous studies have explored spinning preforms to fabricate various types of fibers, including twisted single‐mode fibers,^[^ ^41^ ^]^ helically twisted photonic crystal fibers,^[^ ^42^ ^]^ and fiber‐based probes for brain activity mapping.^[^ ^43^ ^]^ As shown in **Figure** **2**A, a spinning motor is attached to the linear stage, enabling the preform to spin at an adjustable angular velocity during the draw. This approach allows the fabrication of long lengths of multi‐lumen tubing with helical channels, offering pitch sizes ranging from millimeters to tens of centimeters. Furthermore, the versatility of this approach extends to creating both straight and helical lumens by modifying the spinning motor, as demonstrated in Figure 2B,C, which shows multi‐lumen tubing featuring both helical and straight lumens.

To evaluate the performance of our helically routed lumina, we conducted finite element analysis (FEA). Preliminary investigations assessed the friction between the pull wires and the helically routed channels, indicating that helical routing with pitches of 50 and 60 mm minimized friction for one‐meter‐long tubing. Using ANSYS 2020 R2, we developed simplified catheter models with fixed pull‐wires to analyze uncontrolled displacement of the catheter tip (Figure S5, Supporting Information). In these models, the junction between the catheter tip and the shaft was fixed, and both ends of the tendon were secured to the respective ends of the catheter. A displacement of 5 mm was applied to the catheter shaft, resulting in tip displacement due to passive shaft bending. The results showed a significant reduction in maximum uncontrolled displacement at the flexible distal tip, decreasing by 80%, from 1.05 mm to 0.20 mm, when helically routed pull wires were employed. Additionally, bench‐top studies were conducted to evaluate the performance of the helically routed pull wires in compensating for the passive bending of the catheter shaft.

### 2.3 Steerable Catheter System

By implementing the thermal drawing techniques described above, the drawn multi‐lumen tubing with various configurations (Table S1, Supporting Information) was processed and assembled into a steerable catheter. The system comprised two main components: a catheter body with a steerable tip and a manual handle for remote tip control. We employed a tendon‐driven steering mechanism with an MR‐visible tendon serving as the pull‐wire,^[^ ^44^ ^]^ ensuring unobstructed MR visibility from the handle to the distal end. To ensure complete safety, the entire catheter system was constructed using MR‐safe materials.^[^ ^25^ ^]^

**Figure 3:**
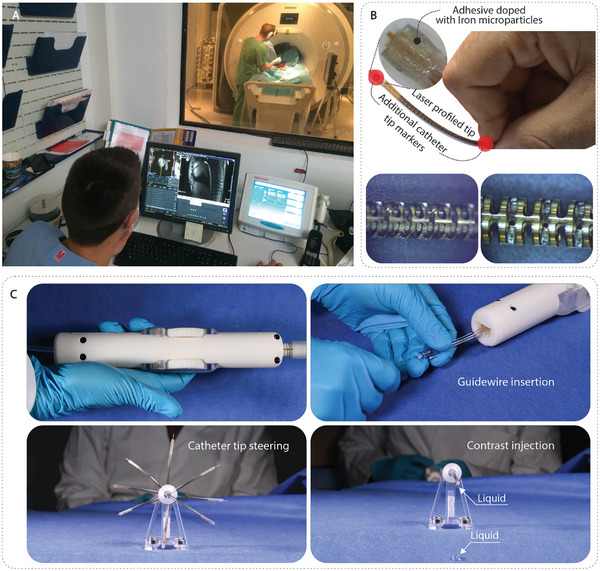
MR‐guided endovascular intervention and detailed views of the steerable catheter system. A) Photograph of an MR‐guided endovascular intervention on a porcine model within the MR suite. B) (upper) Steerable catheter tip with labeled positions of additional tip markers. (lower) Detailed image of laser‐profiled slots at the tip of the steerable catheter. C) Top view of the catheter‐handle assembly, and the basic functions of the assembled steerable catheter system: guidewire insertion from the back of the handle, catheter tip steering (superimposed images of 8 configurations), and contrast (fluid) injection.

The catheter body featured a flexible yet stiff shaft ≈90 cm in length and a more flexible distal end of ≈10 cm. It included a central working channel for other instruments and liquid injection, as well as four peripheral channels for pull wires, enhancing both functionality and visibility. Braid reinforcement was strategically incorporated along the catheter length to enhance mechanical properties such as torquability, whip reduction, and kink resistance without significantly increasing the catheter's dimensions. For the steerable tip (**Figure** **3**B), inspired by Clogenson et al.’s monolithic approach,^[^ ^45^, ^46^ ^]^ we used laser cutting to enhance the flexibility and maneuverability of the distal end. This approach formed 2 DOF perpendicular compliant flexure hinge joints through notches in the tubing.^[^ ^47^ ^]^ The catheter body was then covered with two layers of polyethylene heat‐shrink tubing to encapsulate the braid reinforcement and prevent thrombogenesis. An atraumatic tip made of low‐shore‐hardness platinum‐cured silicone tubing was added, followed by a hydrophilic polymer coating to reduce friction and improve maneuverability. To enhance visibility in MR images, we incorporated passive negative markers with iron microparticles at the distal and proximal tips of the catheter.^[^ ^21^ ^]^

The steering of the catheter's distal end was simplified through an intuitive control method, eliminating the need for complex hardware or software.^[^ ^48^ ^]^ A carefully designed handle served as the interface, enabling remote steering of the catheter tip and performing other functions (Figure 3C). Similar to the ACUSON AcuNav catheter, the 3D‐printed handle comprised two dials positioned on both sides, each controlling the deflection of the distal end in two directions (1 DOF) for four‐way bending. This was achieved through a double‐rack‐and‐pinion mechanism that converted rotary motion into linear motion. As the dial rotated, the pinion and racks moved in opposite directions, tensioning one pull‐wire while releasing the other, resulting in the flexible distal end bending towards the tensioned pull‐wire. The involute contours of the dial provided an improved grip, and the gear‐like profile allowed for seamless integration with other gears, enhancing the handle's potential for direct integration with robotics. A pin vice was mounted at the handle's proximal end for fine‐tuning the tensioning of the pull‐wires during assembly. Additionally, a universal Luer lock port was positioned at the rear of the handle to facilitate fluid injection and the insertion of a micro guidewire into the catheter's central lumen.

### 2.4 Multi‐Lumen Tubing with Wire Feeding

For the Tiger‐shaped active tracking catheter, we exploited another unique capability of thermal drawing. Through the fusion of the thermal drawing process with a feeding mechanism, wires can be integrated into the fiber,^[^ ^35^, ^49^ ^]^ a task often considered challenging with similar mass production techniques. As illustrated in Figure 2C, the wire is threaded through pre‐designed channels within the cross–sectional structure of the preform and co‐fed during the drawing process. As the channels constrict along with the entire cross–sectional configuration, the fed wire becomes securely anchored within the fiber. For a detailed visualization of the co‐feeding procedure, please refer to Video S7 in the Supplementary Materials of reference [34].

### 2.5 Steerable Catheter System

The Tiger‐shaped catheter, composed of PC material, yields poor MR imaging visualization, necessitating the integration of an active tracking system to enhance visibility, particularly at the catheter tip. This active tracking system consists of a receive radiofrequency (RF) coil resonant at the Larmor frequency of the MRI system (128 MHz at *B*_0_ = 3 Tesla). Specifically, a single‐loop coil (18 × 1.6 mm^2^) etched from 35 µm copper on a 50 µm polyimide (Kapton) was added to the tip of the catheter (Figure 8C). This coil has been successfully used in previous studies of MR‐guided coronary catheterization.^[^ ^21^, ^50^, ^51^ ^]^ The coil requires an electrical connection via a coaxial cable to the MR receiver system.^[^ ^52^, ^53^ ^]^ Typically, when adding a coaxial cable to a commercially available catheter, the cable is either pulled through an open lumen of the catheter,^[^ ^50^, ^51^, ^53^ ^]^ or placed alongside the catheter using thin shrink tubing.^[^ ^52^ ^]^ These methods can lead to cable breakage or poor connections. Thus, a fully integrated manufacturing process that does not exert strong forces on the cable is always beneficial.

During the fabrication of the Tiger‐shaped catheter, the coaxial wire, with a diameter of 0.2 mm, was co‐fed into the smaller channel of the preform, resulting in a fiber with an embedded wire. This integration eliminated the need for the coaxial wire's outer cladding, reducing the overall diameter of the catheter and producing a more compact and durable design.

### 2.6 Bench‐top Mechanical Characteristics for Steerable Catheters

**Figure 4:**
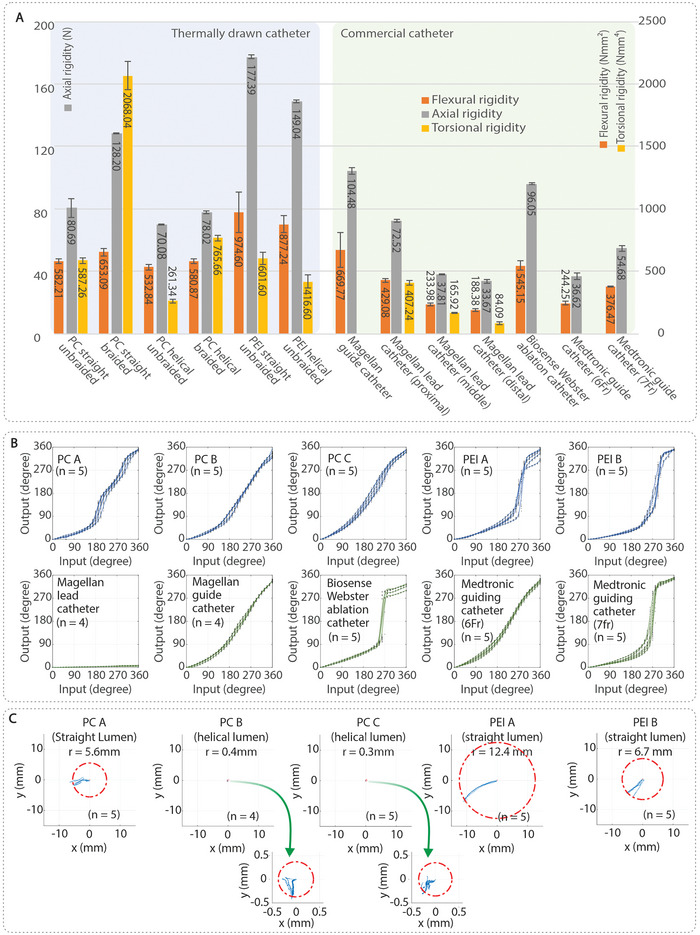
Mechanical characterization results for the steerable catheter. A) Flexural rigidity (Experiment 1‐1; *n =* 5), axial rigidity (Experiment 2; *n =* 4 for distal section of Magellan lead catheter, Medtronic guide catheter, PC helical braided catheter, and PC straight unbraided catheter, *n =* 3 for all others), and torsional rigidity (Experiment 3‐1; *n =* 5) of the catheter shafts. B) Catheter shaft's angular response under rotary input (Experiment 3‐2). C) Uncontrolled deflection of the catheter tip resulting from a 90° catheter shaft deflection (Experiment 5).

To thoroughly evaluate the mechanical performance of our thermally drawn catheter shafts in comparison to commercially available counterparts, we conducted a comprehensive set of experiments focused on key characteristics: flexibility (flexural rigidity), pushability (axial rigidity), and torquability (torsional rigidity). The results from these experiments—flexural rigidity (Experiment 1), axial rigidity (Experiment 2), and torsional rigidity (Experiments 3–1)—were integrated and compared in **Figure** **4**A. Specific details of the catheter shaft samples are provided in Tables S1–S3 (Supporting Information). Custom‐designed setups were used for testing, and the performance of the assembled catheter handle system was also evaluated in terms of torque response (Experiment 3‐2), steerability (Experiment 4), and the suppression effect of helically routed pull‐wires on undesired motion of the flexible tip during passive bending (Experiment 5).

Flexibility reflects the stiffness of the catheter when subjected to bending, which is crucial for guiding and supporting endovascular devices while maintaining distal flexibility. The detailed results from these experiments are displayed in Figure S6A,C (Supporting Information). Experiment 1‐1 involved bending the catheter shafts and measuring the force required. Under dry conditions, most of our in‐house fabricated catheter shafts exhibited higher flexural rigidity than commercial catheters. After immersion in water (Experiment 1–2), the flexural rigidity of all tested catheters decreased, but our catheters maintained higher flexural rigidity compared to their commercial counterparts. Stress relaxation over time (Experiment 1–3) was observed, with Medtronic Guiding Catheters experiencing the largest drop (averaging 22.5%), while our PC catheter shafts with helical lumina showed the smallest drop (averaging 8.10%).

Pushability, which measures the catheter's ability to transmit forces in the axial direction, was assessed in Experiment 2, with results presented in Figure S7A,D (Supporting Information). Catheters with higher flexural rigidity demonstrated superior axial force transmission. The PEI catheter shafts exhibited the highest axial rigidity, ranging from 149.07 N to 177.39 N. Over‐braiding increased axial rigidity by 37.06% for parallel lumina and 10.17% for helical lumina in PC catheter shafts. With the exception of the braided PC catheter shaft with parallel lumina, our in‐house fabricated catheters displayed pushability comparable to commercial catheters.

Torquability was investigated in Experiments 3‐1 and 3‐2. In Experiment 3‐1, samples were twisted around their axis using a servo motor. Catheter shafts with parallel lumina exhibited higher torsional rigidity compared to helical lumina shafts. Detailed results are presented in Figure S8A (Supporting Information). Braiding improved torsional rigidity by ≈71.6% for parallel lumina and 65.9% for helical lumina. Some commercial catheters experienced kinking due to thin walls (Figure S8C, Supporting Information). In Experiment 3‐2, the torque response of the distal end was examined in passively bent catheters, simulating the transfemoral trajectory of the catheter across the aortic arch. As illustrated in Figure 4B, catheter shafts with higher flexural rigidity showed poor linear torque transmission, resulting in catheter whip motion. PC‐based catheters and certain commercial catheters demonstrated smoother and more linear torque transmission.

The steerability of integrated catheter‐handle assemblies was evaluated in Experiment 4‐1, assessing the workspace and positioning accuracy of the catheter, and in Experiment 4‐2, evaluating the steering performance after passive bending. The results of Experiments 4‐1 and 4‐2 are graphically illustrated in Figures S13–S28 (Supporting Information). The catheters demonstrated good repeatability, with standard deviations of less than 1 mm for most samples, similar to what has been reported in previous studies,^[^ ^54^ ^]^ and <5 mm overall. Backlash, indicating the difference in positioning between forward and backward steering, was observed in all samples. Catheters with straight lumina had an average backlash of ≈5 mm, while the catheter with helical lumina exhibited a larger backlash of ≈10 mm. Backlash increased when straight lumina catheters were bent, but this was not observed in the helical lumina catheter. The torques required to turn the knob fell within the range of ± 0.02 Nm, indicating the forces involved in catheter deflection and assembly friction.

Experiment 5 tested the effectiveness of helically routed pull‐wires in suppressing passive deflections. As shown in Figure 4C, the helical lumina catheters demonstrated significantly smaller deflections compared to parallel lumina catheters, ranging from 0.3 mm to 0.4 mm versus 5.6 mm to 12.4 mm, respectively. These differences in deflection among the parallel lumina samples can be attributed to diameter variations. For instance, PEI A, with a larger outer diameter (2.7 mm to 2.88 mm), displayed greater deflections compared to PC A and PEI B, which had outer diameters ranging from 2.41 mm to 2.55 mm. It is important to note that larger tip deflections can negatively impact catheter steering accuracy and potentially cause harm to surrounding tissues and vessel walls.

### 2.7 In vitro MR Compatibility and Phantom Study for Steerable Catheter

**Figure 5:**
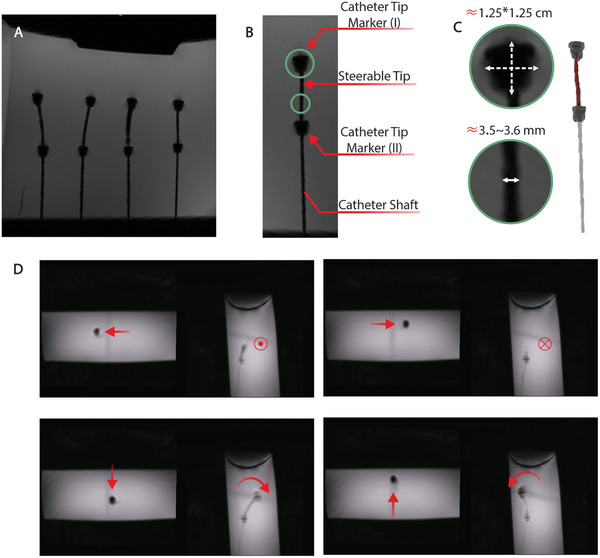
In vitro MRI test for steerable catheter. A) Imaging artifacts of four steerable catheters acquired using a 3D Flash sequence at 3 Tesla. B) Labeling of the artifacts and inset of the catheter tip marker artifacts (upper) and catheter shaft artifacts (lower) with dimensions. C) Reconstructed 3D model of the steerable catheter from the acquired scan. D) Real‐time imaging in the transverse and sagittal planes as the catheter is steered in all four directions.

The mechanical efficacy and MR visibility of the steerable catheter were assessed in various simulated clinical scenarios. To evaluate MR visibility, four catheters were placed in a water‐filled container, and high‐resolution MR images were acquired using a clinical 3‐Tesla MR system with a 3D FLASH sequence. The resulting images (**Figure** **5**A–C) clearly displayed the catheter shaft and tip markers, producing artifacts comparable in size to those observed in previous studies with guidewires.^[^ ^44^ ^]^ The shaft artifacts had a mean diameter of 2.0 ± 0.2 mm, while the tip marker measured ≈12.5 mm by 12.5 mm. Notably, the inclusion of four MR‐visible pull‐wires for steering did not significantly impact the size of the artifacts. On average, the shaft artifacts had a thickness of 3.55 mm, ensuring excellent visibility in MR images.

To evaluate the catheter's four‐way steering capability, we employed two projected planes that provided simultaneous visualization of a transverse plane at the distal‐most point of the catheter tip and a frontal plane spanning the entire catheter length. This imaging approach allowed us to observe and document the catheter's precise movements in different directions, including left, right, up, and down. The results (Figure 5D; Movie S1, Supporting Information) vividly showcased the catheter's exceptional maneuverability and its ability to navigate challenging anatomical pathways. Additionally, post‐processing provided a comprehensive illustration of the catheter tip's steering capability in all four directions, offering a valuable tool for real‐time guidance during procedures.

**Figure 6:**
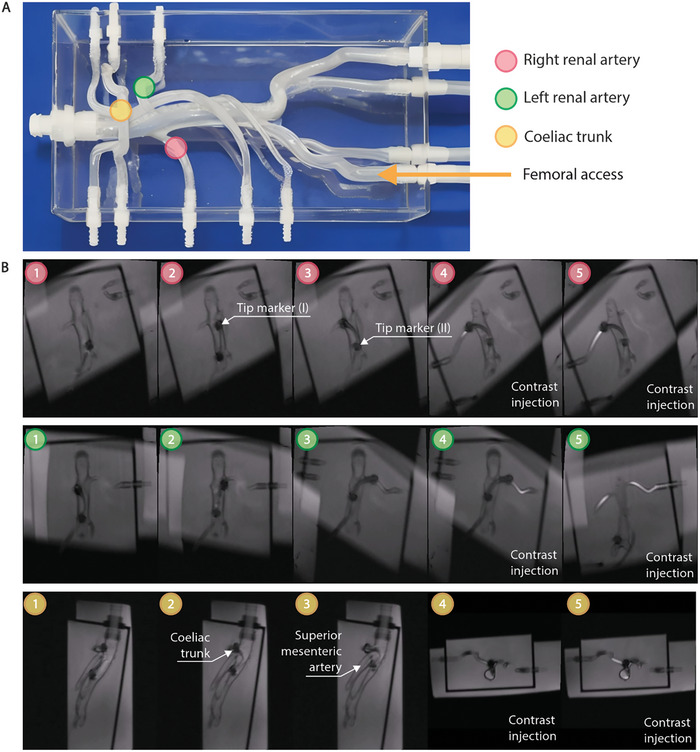
In vitro phantom study for the steerable catheter. A) Abdominal phantom with left and right renal artery (LRA and RRA) and coeliac trunk artery target targets. B) Image sequence illustrating probing of the LRA (green), RRA (red), and the coeliac (yellow) in an abdominal phantom using the steerable catheter under real‐time MR guidance.

To validate the clinical applicability of our steerable catheter, phantom experiments were conducted using a soft silicone vascular phantom resembling a normal adult abdominal aorta. The phantom was filled with water, and under real‐time MRI guidance, a novice operator successfully maneuvered the catheter into three primary target vascular structures: the right renal artery (RRA), left renal artery (LRA), and coeliac trunk. As shown in **Figure** **6** and Movie S2 (Supporting Information), the probing procedure was further confirmed using contrast agent injection for enhanced visualization. These results highlight the feasibility and efficacy of our steerable catheter in navigating complex anatomical structures, even for operators with limited experience. Preliminary results from similar experiments are available in previous studies.^[^ ^33^ ^]^

### 2.8 In vivo Animal Study for Steerable Catheter and Active Tracking Catheter

**Figure 7:**
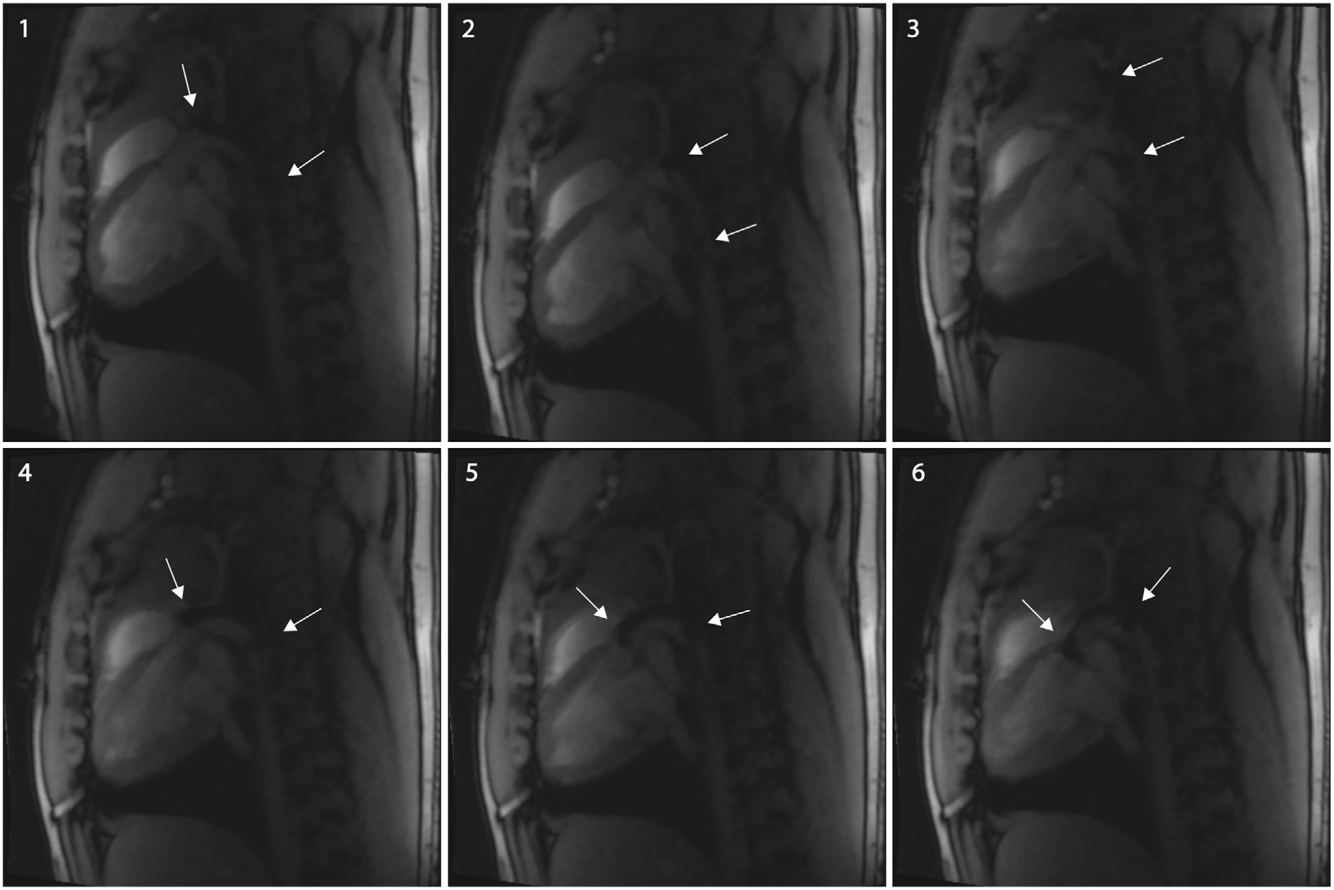
In vivo real‐time MRI of steerable catheter manipulation in a clinical 3 Tesla system. Arrows point at the two markers on the catheter tip. 1) Catheter advancing towards the aortic arch. 2,3) Catheter pointing towards the carotid artery. 4,5) Catheter steering towards the aortic arch. 6) Catheter crossing the aortic arch towards the aortic root.

To validate the clinical feasibility and performance of our steerable catheter, we conducted in vivo testing using a porcine model under general anesthesia. Real‐time MRI was employed to monitor the catheter's position during the intervention, ensuring accurate guidance throughout the procedure. The cardiologist relied on a non‐magnetic and RF‐shielded in‐room screen for real‐time visualization of the catheter (Figure 3A). Utilizing a pre‐implanted femoral access sheath (10F), the catheter was successfully introduced into the arterial system. MRI displayed both the anatomy and the catheter, offering comprehensive coverage of the vascular system. Remarkably, the maneuverability of our catheter was effectively demonstrated by successfully bending the catheter tip in the ventral direction to enable the crossing of the aortic arch towards the heart (**Figure** **7** and Movie S3, Supporting Information). Throughout the study, we adhered to the guidelines of the local ethics committee and regional council, ensuring the utmost care and compliance with animal welfare standards. These promising results underscore the potential of our steerable catheter to revolutionize medical interventions and contribute to improved patient outcomes.

**Figure 8:**
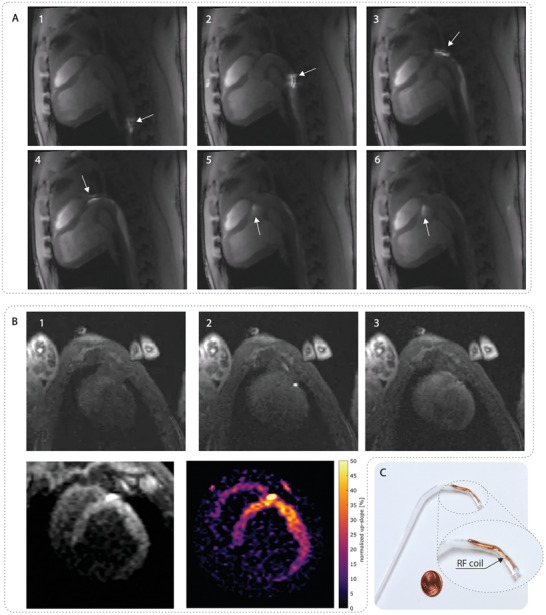
In vivo real‐time MRI of active tracking catheter and photograph of tip details. A) Catheter crossing the aortic arch with arrows pointing at the catheter tip. B) Intracoronary injection of gadolinium‐containing contrast for selective perfusion, enabling semi‐quantitative mapping by normalizing values to the up‐slope in the left anterior descending artery. C) Active tracking catheter tip with labeled positions of RF coil.

Similar experiments were performed in a porcine model using an active tracking catheter. As shown in **Figure** **8** and Movie S4 (Supporting Information), the catheter is clearly visible under real‐time magnetic resonance imaging. Both the location and shape of the catheter tip are depicted by the bright signal from the loop coil. In addition, the shaft of the catheter is visible as a hyperintense signal that is picked up by the coaxial cable. Using the active tracking catheter, the left coronary artery (LCA) was successfully intubated. This allowed for a selective perfusion measurement of the myocardial segments supplied by the LCA. Therefore, a gadolinium‐based contrast agent was injected via the catheter under real‐time imaging in a mid‐ventricular short‐axis view. The injection resulted in a selective signal enhancement in the septal, anterior, and lateral segments of the left ventricle demonstrating the successful intubation of the LCA. It is worth noting that the active tracking catheter, though not MR‐safe by definition has never caused any adverse effects during more than 30 procedures.

## 3 Discussion and Conclusion

The versatility and precision of thermal drawing technology have been recognized across various industries, particularly for its ability to produce fibers with precise cross–sectional geometries. However, its application in the medical field, especially in fabricating surgical instruments with high aspect ratios, remains largely unexplored. This study demonstrated the feasibility of employing thermal drawing technology to manufacture catheters specifically designed for MR‐guided endovascular interventions. Compared to the commonly used extrusion method, which can be complex and costly, thermal drawing offers a cost‐effective and rapid alternative, eliminating the need for additional complex tooling or manufacturing processes. Furthermore, our thermal drawing approach provides benefits beyond rapid tubing fabrication, enabling the seamless integration of helical lumina structures and wire feeding in a single step‐ difficult to achieve with traditional approaches.^[^ ^34^ ^]^

Remarkably, the entire fabrication process for the two catheter systems was completed within a few weeks at our research facility. The primary costs were limited to polymers and machine operational expenses, showcasing a rapid and economical transition from concept to prototype. This achievement aligns with Technology Readiness Level 3 (TRL 3), according to UK Research and Innovation (UKRI), which involves analytical and experimental proof‐of‐concept for critical functions or characteristics. The rapid development process highlights thermal drawing's potential to expedite innovation in medical device manufacturing, particularly for applications requiring intricate geometrical configurations and specific material properties.

The versatility of thermal drawing technology allows for tuning the catheter's mechanical properties by manipulating various factors, including the selection of different materials with different mechanical characteristics. Depending on the material, preform fabrication can be achieved through direct machining of polymer bars or advanced techniques like 3D printing^[^ ^55^ ^]^ and molding to create preforms with complex geometries. This flexibility also allows the integration of multiple materials with different thermal and mechanical properties into a single preform, which is then thermally co‐drawn to achieve the desired dimensions.^[^ ^31^, ^56^ ^]^ During the drawing process, cross–sectional dimensions can be dynamically adjusted by manipulating the drawing speed, enabling the production of components with tailored dimensions to meet specific design requirements. However, achieving the desired fiber diameter necessitates careful temperature regulation during the heating phase and precise control of the drawing velocity.

Since MR‐guided endovascular intervention technology has not yet been widely adopted, there is no well‐established gold standard for assessing the performance of instruments in such interventions. The experimental settings, choice of critical mechanical properties, and number of repetitions were designed based on related works.^[^ ^57^, ^58^, ^59^, ^60^, ^61^ ^]^ Our in‐house fabricated catheter shafts, designed for MR‐guided endovascular interventions, exhibit mechanical properties comparable to their commercial counterparts, reaffirming the effectiveness of our manufacturing approach.

Both in vitro and in vivo experiments confirmed that the helical lumen structure for the steerable catheter effectively compensates for undesired motion caused by the tendon‐driven mechanism. However, the helical luminal design also increases tendon travel distance, leading to heightened friction. Despite efforts to eliminate backlash, achieving complete avoidance remains challenging. To ensure visibility along the catheter's entire length, passively MR‐visible MaRVis rods were employed as pull‐wires for steering.^[^ ^62^ ^]^ These rods provide sufficient pulling force for steering the catheter tip, although they are prone to kinking when subjected to pushing forces. Passive markers being inherently MR‐safe provide visible artifacts that indicate the two ends of the steerable section without significantly compromising the visibility of surrounding anatomical structures.

Active markers provide a strong positive signal at marker locations. Combining the catheters with an active tracking RF coil proved highly beneficial as it allowed for the visualization of the catheter tip with positive contrast during the intervention. Positive contrast is essential for identifying the tip position and catheter orientation, both of which are crucial for successfully advancing a catheter into branching vessels such as coronary arteries.^[^ ^51^ ^]^ However, active tracking, which uses coaxial cables to guide the MRI signal from the tracking coil to the receiver poses risks of device heating, especially when the device length in the MRI approaches the resonance length in tissue. Several methods have been proposed to mitigate this risk, such as integrated baluns or transformers‐ although these additional structures require additional wiring along the catheter.^[^ ^63^, ^64^ ^]^ Current fabrication approaches involve pulling cables through a dedicated open lumen of the catheter or placing them alongside the catheter using shrink tubing. These approaches not only increase the complexity of manufacturing but also increase the size of the catheters. Our platform enables compact integration by embedding wires into the catheter walls during the drawing process, reducing the need for post‐processing steps. This integration eliminates the necessity for additional lumens for wiring and external plastic insulation for cables, thereby enhancing both compactness and durability. Moreover, our platform holds the potential for integrating prefabricated complex electronic structures directly into the catheters during the drawing process,^[^ ^49^, ^65^ ^]^ further minimizing the need for extensive post‐processing steps and reducing catheter dimensions. This capability could be particularly valuable in the development of more advanced, multifunctional catheters.

Overall, this work highlights the potential of thermal drawing technology as a valuable tool for medical device fabrication. By offering versatility in material integration and design flexibility, this cost‐effective and highly customized fabrication approach holds great potential, empowering researchers and companies with increased flexibility to explore a wide range of designs and modifications without incurring extensive investments or prolonged production timelines.

## 4 Experimental Section

### Multi‐Lumen Tubing

Both PC and PEI polymers were selected as the material for the steerable catheters, using medical/life science grade (LSG) polymer rods (Mitsubishi Chemical Advanced Materials UK Ltd., Lancashire, United Kingdom) for the manufacture of the preform. Precise placement of the through‐holes in the preform was ensured through peck drilling with a driller of 6 mm. The thermal drawing process began by feeding the preform into the draw tower's three‐zone tube furnace (TV‐05 Solid Tubular Furnace, The Mellen Company, USA) at a downfeed speed of *v_DF_* = 1 mm min^−1^. The preheating temperature in the top zone was set to 140 °C for PC and 220 °C for PEI; the middle zone temperature was adjusted above the glass transition temperature of PC (*T_g_* = 145 °C, set temperature = 240 °C) and PEI (*T_g_* = 215 °C, set temperature = 340 °C); bottom zone temperatures were set at 85 °C for PC and 95 °C for PEI. The fiber was monitored using a laser micrometer (Laserlinc, USA) to measure the outer diameter and a three‐wheel tension sensor to measure its tension, which was ideally maintained at ≈100 g. The draw speed (*v_D_*) was determined by applying the conservation of mass equation.

(1) $$A_{P}\times v_{DF}=A_{F}\times v_{D}$$

Where *A_P_* and *A_F_* were the cross–sectional area of the preform and the fiber.

The selected material for the active tracking catheter shaft was PC. The cross–section of the preform, depicted in Figure S1B (Supporting Information), was prepared by drilling two holes (28.5 and 3 mm) into a PC rod. During the drawing process, temperatures were set to 140, 240, and 85 °C across the three zones, with *v_DF_* = 1 mm min^−1^ and *v_D_* = 225 mm min^−1^, ensuring a consistent fiber diameter of 2.5 mm. A customized wire feed mechanism, featuring a wire spool and pulley, was used to guide and passively feed the wire of diameter 0.2 mm through the preform during drawing. As the preform was drawn, reducing the channel diameter, the wire was integrated into the fiber, allowing for the incorporation of metal wires using the existing fiber drawing platform.

The tip of the active tracking catheter was a single‐lumen tubing made from COCe (E‐140, TOPAS Advanced Polymer GmbH, Germany) through the thermal drawing. The preform (inner diameter = 32 mm, outer diameter = 40 mm) was created through vacuum compression molding,^[^ ^35^ ^]^ at a heating temperature of 190 °C, pressure of 0.2 MPa, and time duration of 20 hours. After drawing (middle zone temperature = 210 °C, *v_DF_* = 1 mm min^−1^, *v_D_* = 256 mm min^−1^), the resultant single‐lumen tubing had an inner diameter of 2 mm and outer lumen of 2.5 mm.

For detailed information on the preform fabrication and fiber drawing procedure, please refer to Notes S1,S2 in Supporting Information.

### FEA of Helical Lumen Catheter

FEA was carried out to investigate the uncontrolled tip steering caused by the 100 mm shaft being passively deflected by 5 mm (ANSYS 2020 R2). The tip and shaft material were set to PC with the following physical/mechanical properties: density = 1200 kg m^−3^, Young's modulus = 2380 MPa, Poisson's ratio = 0.399, and tensile yield strength = 6.21 MPa. On the other hand, the pull wire was set to stainless steel with the following physical/mechanical properties: density = 7500 kg m^−3^, Young's modulus = 1.93e5 MPa, Poisson's ratio = 0.31 and tensile yield strength = 207 MPa. The pulling wire was fixed at both ends of the catheter and the intersection surface between the proximal end of the tip and the distal end of the shaft was also fixed. A passive offset of 5 mm was applied midway (50 mm) from the proximal end of the shaft.

### Post‐processing of Catheters

Post‐processing steps were conducted to enhance the mechanical properties and functionality of the drawn multi‐lumen tubing. For steerable catheters, over‐braiding improved torqueability and kink resistance, while laser cutting the distal ends increased the flexibility of the tip. The catheter tips were encapsulated with a PTFE tube and the shafts were coated with hydrophilic coating to reduce thrombogenesis and improve maneuverability, with passive negative markers added for MR visibility. For active tracking catheters, a Tiger‐shaped tip was formed using a 3D‐printed mold. A resonance coil was attached and connected to a coaxial wire within the catheter shaft for active MRI tracking. The COCe tip and PC shaft were bonded using a PTFE shrinking tube for a secure connection. Materials and detailed methods were available in Notes S3,S4 in Supporting Information.

### Integration of Steerable Catheter and Handle

Details of the steerable catheter assembly are depicted in Figure S3 (Supporting Information). For visibility under MR images, MaRVis rods^[^ ^62^ ^]^ were utilized as pull‐wires within the catheter. Most of the catheter components were printed from a biocompatible Acrylonitrile Butadiene Styrene (ABS) using a Fused Deposition Modelling (FDM) printer (FORTUS 400mc, Stratasys Ltd., Eden Prairie, MN, USA). Plastic screws (PEEK and Glass Reinforced Resin Screws, Misumi Europa GmbH, Frankfurt, Germany), bearings (Linear Plain bearing GSM‐0608‐04, Igus, Northampton, United Kingdom), and the pinion (0.5 Mod × 13 Tooth Metric Spur Gear In. Hostaform, Bearingboys, Norwich, United Kingdom) were excluded from the printing process.

The integration of the catheter involved inserting the catheter into the handle through a pin vice, passing a series of guide channels to suppress its lateral motion and a pull‐wire routing block that prevents crosstalk of the pull‐wires and friction‐caused damage. The shaft was secured in place by tightening the vice nut. Four polytetrafluoroethylene (PTFE) tubing (0.4 × 0.6, Iwase, Kanagawa, Japan) were threaded through the pull‐wire routing block, extending through skived side holes that provide access to the four lateral channels of the shaft. Pre‐lubricated pull‐wires were fed through the PTFE tubing guide and advanced through the distal end of the catheter. Segments of fused silica capillary (TSP450670, CM Scientific, Keighley, United Kingdom) acting as stoppers were affixed using cyanoacrylate glue (Loctite 416, Henkel, Dusseldorf, Germany) at the tendon's distal end. The proximal ends of the pull‐wires were equally tensioned and secured to their corresponding racks using a combination of a locking screw and Loctite 416. The racks were then held in place by the knob/pinion assembly, which was mounted to the handle using an external cover embedded with a mechanical lock. To minimize backlash, the pull‐wire routing block was fixed using hot melt adhesive, effectively preventing unintended displacement of the pull‐wires. Finally, a Luer lock port was connected at the proximal‐most point of the catheter shaft.

### Benchtop Experimental Setups

In order to assess the mechanical characteristics of the catheter shaft, including flexibility (Figure S6, Supporting Information), pushability (Figure S7, Supporting Information), torqueability (Figures S8 and S9, Supporting Information), steerability (Figure S10 and S11, Supporting Information), and the suppressive effect of the helical structure on undesired motion (Figure S12, Supporting Information), a series of experiments were conducted using the custom‐designed experimental setups. Materials and methods were available as Note S5–S9 in Supporting Information.

### In Vitro and In Vivo Test

All MRI experiments were performed on a clinical 3‐Tesla system (Magnetom Prisma, Siemens). The visibility of the steerable catheter was first tested using a 3D FLASH sequence with the following parameters: echo time (TE), 2.2 ms; repetition time (TR), 5.0 ms; flip angle (α) = 10°; field of view (FoV), 300 × 300 × 50 mm^3^; matrix size, 240 × 240 × 40; bandwidth (BW), 400 Hz/pixel. MR images were acquired for four catheter samples immersed in water under these settings. The bending of one steerable catheter tip was imaged in real‐time imaging using a 2D bSSFP sequence acquired in two orthogonal planes under settings of TE/TR = 1.3/3.0 ms, α = 43°, BW = 930 Hz/pixel, FoV = 300 × 56 mm^2^, matrix size = 192 × 138, slice thickness (SL) = 15 mm.

A silicon phantom in the shape of a normal adult abdominal aorta (Elastrat Sarl, Geneva, Switzerland) was used for the accessibility investigation. Probing of the right and left renal arteries, as well as the coeliac trunk was imaged in real‐time using a 2D FLASH sequence (TE/TR = 2.0/4.9 ms, TA/image = 251 ms, α = 11°, BW = 500 Hz/pixel, FoV = 300 × 256 mm^2^, matrix size = 192 × 138, SL = 10 mm). To verify the successful intubation of the individual arteries, a bolus of diluted gadolinium contrast agent was injected through the catheter.

The steerable catheter and the Tiger‐shaped active tracking catheter were tested in vivo in two juvenile domestic landrace pigs. The experimental protocols received approval from the local ethics committee of Freiburg University and the regional council of Freiburg, Baden‐Wuerttemberg, Germany (license numbers 35–9185.81/G‐15/156 and 35–9185.81/G‐16/78) and the experiments were conducted in accordance with FELASA and GV‐SOLAS standards for animal welfare. The animals were anesthetized and mechanically ventilated throughout the experiments. A 10F arterial access sheath was introduced into the right femoral artery via ultrasound guidance prior to the MR exam. The animals were positioned in head first supine position with the heart at the iso‐center and the 32‐channel spine coil as well as an anterior 18‐channel coil array were used for signal reception. Navigation of the catheters was guided using a real‐time FLASH sequence (TE/TR = 1.3/3.4 ms, α = 10°, FoV = 289 mm^2^, matrix size = 192 × 144, BW = 790 Hz/pixel, SL = 8 mm). First, the steerable catheter was tested in one animal. Therefore, the catheter advanced towards the aortic arch in a straight configuration. Then, the tip was intentionally bent towards the ventral direction to enable further advance of the catheter across the ascending aorta to the aortic root. The active tracking catheter was tested in the second animal. The catheter was advanced over a 0.018″ guidewire (Terumo) until the catheter tip was positioned in the ascending aorta. Then, the guidewire was removed, and the catheter tip was maneuvered into the LCA.

The successful intubation was verified by performing a selective perfusion measurement. Therefore, a bolus of 1% gadolinium solution was injected via the catheter during imaging of the short‐axis of the heart with an inversion recovery FLASH sequence (TE/TR = 1.0/2.0 ms, TI = 95 ms, α = 10°, FoV = 360 × 70 mm^2^, matrix size = 192 × 106, BW = 1185 Hz/pixel, SL = 8 mm, single breath‐hold).

### Statistical Analyses

All data were collected and calculated using MATLAB. Data were presented as mean ± standard deviation.

### Ethical Statement

The experimental protocols received approval from the local ethics committee of Freiburg University and the regional council of Freiburg, Baden‐Wuerttemberg, Germany (license numbers 35–9185.81/G‐15/156 and 35–9185.81/G‐16/78) and the experiments were conducted in accordance with FELASA and GV‐SOLAS standards for animal welfare.

## Conflict of Interest

The authors declare no conflict of interest.

## Author Contributions

M. E. M. K. Abdelaziz and L. Tian contributed equally to this work. M.E.M.K. Abdelaziz was responsible for the conceptualization, methodology, investigation, visualization, and writing of the original draft. L.T. also contributed to the conceptualization, methodology, investigation, visualization, and writing of the original draft. B.T. handled conceptualization, methodology, funding acquisition, project administration, supervision, and writing through review and editing. E.Y. focused on funding acquisition, while T.L., S.R., M.B., T.H., A.M., C.V.Z.M., and K.D. all contributed to the methodology. Additionally, S.R. and M.B. assisted with writing, review, and editing, and G.Z.Y. was responsible for funding acquisition.

## Supporting information



Supporting Information

Supplemental Movie 1

Supplemental Movie 2

Supplemental Movie 3

Supplemental Movie 4

## Data Availability

The data that support the findings of this study are available in the supplementary material of this article.
