# Supplementary material for: Thermally Drawn Polymeric Catheters for MR‐Guided Cardiovascular Intervention
Source: Adv Sci (Weinh). 2024 Oct 15;11(45):2407704. doi: 10.1002/advs.202407704 (PMC11615795; doi:10.1002/advs.202407704)
Supplement: Supplementary file 1 — Supporting Information [file ADVS-11-2407704-s001.pdf]

ADVANCED SCIENCE
Open Access

# Supporting Information

for *Adv. Sci.*, DOI 10.1002/advs.202407704

Thermally Drawn Polymeric Catheters for MR-Guided Cardiovascular Intervention

*Mohamed E. M. K. Abdelaziz, Libaihe Tian, Thomas Lottner, Simon Reiss, Timo Heidt, Alexander Maier, Klaus Düring, Constantin von zur Mühlen, Michael Bock, Eric Yeatman, Guang-Zhong Yang and Burak Temelkuran**

# Supporting Information

## Thermally Drawn Polymeric Catheters for MR-Guided Cardiovascular Intervention

*Mohamed E. M. K. Abdelaziz, Libaihe Tian, Thomas Lottner, Simon Reiss, Timo Heidt, Alexander Maier, Klaus Düring, Constantin von zur Mühlen, Michael Bock, Eric Yeatman, Guang-Zhong Yang, Burak Temelkuran**

Corresponding author: b.temelkuran@imperial.ac.uk

**The PDF file includes:**

Notes S1 to S9

Figure S1 to S28

Table S1 to S5

Legends for Movie S1 to S4

**Other Supplementary Materials for this manuscript include the following:**

Movie S1 to S4

**Notes S1: Preform fabrication**

Preforms for steerable catheters were fabricated from commercially available PC and PEI rods, cut to lengths of 140 mm. Due to drill bit length limitations, through-holes were drilled from both ends of each rod (Figure S1C). Drilling points on both ends were precisely marked with a scribe to ensure accuracy. The rods were then secured in place using a V-block fixture to ensure stability during the drilling process. To enhance precision and dimensional accuracy, the holes were peck drilled using a bench drilling machine. After drilling one side, the process was repeated on the opposite side to complete the through holes. Although we did not observe any significant misalignment, any minor discrepancies that may occur are confined to very short segments near the interfaces. These segments can be easily excluded in subsequent fabrication steps.

COCe preforms for the tip of the active tracking catheter were created using vacuum compression molding. A customized stainless-steel mold (Figure S1E) was employed to shape the preforms with an inner diameter of 32 mm and an outer diameter of 40 mm. COCe polymer pellets were loaded into the mold, and a weight was placed atop the molds to exert a pressure of 0.2 MPa. The heating temperature was set to 190°C, and a vacuum was maintained throughout the 20-hour heating period. To compensate for gaps between the pellets, additional pellets were added during the process to ensure uniformity and maintain the structural integrity of the preforms.

**Notes S2: Thermal drawing procedure**

The thermal drawing process employs a multi-zone heating furnace to regulate the temperature profile during the drawing. Each zone – all measuring 100 mm - is designed to provide sufficient time and space for the thermal transitions during the drawing. The temperature of each zone is independently adjustable to serve specific functions, defining an optimal temperature profile that determines the preform-to-fiber geometry.

The first (top) zone is set for preheating the preform to a temperature close to its glass transition temperature ($T_g$) or melting temperature ($T_m$). This preheating ensures uniform heating of the preform, preparing it for the subsequent stage. The second (middle) zone is the most critical zone in the process. It heats the polymer to a viscosity range between $10^4$ to $10^6$ Pa*s, [66] enabling it to stretch without breaking. The third (bottom) zone cools or 'quenches' the polymer, aiming to fix the dimensions and cross-sectional features of the drawn fiber. While this three-zone design is effective, it is not restrictive. The size of the zones depends on the size of the preforms typically drawn. More or fewer zones can be used depending on

specific requirements. This flexibility ensures that the thermal drawing process can be optimized for various polymer materials and preform sizes.

The specific settings for the materials used in this study are summarized in table S5.

The upper end of the preform was connected to a mandrel, which was attached to a linear stage to control its position and feeding rate. The preform was placed in the furnace so that approximately 50 mm at the bottom was in the middle heating zone. A suitable weight (as specified in table S5) was hung from the bottom of the preform to provide the initial pulling force. The furnace temperature was set according to the values in table S5. After a heating period of 30 to 60 minutes, determined by the material's thermal conductivity and the preform’s thickness, the preform began to be drawn into a thin multi-lumen tubing under the traction of the weight. The weight was then removed, and the end of the fiber was connected to a rotating capstan to maintain a constant drawing speed ($v_D$). Concurrently, the mandrel was down feeding at a constant rate ($v_{DF}$) to produce a continuous, uniform fiber. A laser measurement system below the furnace monitored the fiber thickness in real-time, allowing for adjustments to the $v_D$ and $v_{DF}$ as necessary.

**Notes S3: Post-processing of steerable catheter**

After being drawn, segments of the multi-lumen tubing were selected and cut into the desired length. Post-processing was conducted to further enhance the properties of the tubing (Figure S2). To enhance the mechanical properties of the thermally drawn multi-lumen tubing, additional braid reinforcement was added along its length, excluding the distal end. This reinforcement improved torqueability and enhanced kink resistance without significantly increasing wall thickness or outer diameter, achieving the mechanical characteristics of thick-walled catheters with thinner walls. The over-braiding process intertwined synthetic fibers, Kevlar®49 yarns (DuPont, Delaware, United States), using a vertical Maypole type braider (Herzog RU 2/16-80, HERZOG GmbH, Germany). Sixteen spools of these fibers were loaded onto carriers and interlaced in a 1/1 diamond pattern [67] over the tubing (Figure S2A).

After braiding, the non-braided distal end of the tubing was laser cut to increase flexibility (Figure S2B), facilitating the steering of the catheter tip and its atraumatic introduction into tortuous vessels. The design, featuring 2 DOF perpendicular compliant flexure hinge joints, was optimized based on the review article by Jelínek et al.[68] and computational analysis using detasFLEX.[69] Laser micromachining was performed using an LML-femto2000 ultrafast laser micromachining tool (Laser Micromachining, St. Asaph, United Kingdom) with a 343 nm ultraviolet femtosecond laser (Pharos SP-HP, Light Conversion, Vilnius, Lithuania).

The multi-lumen tube was fixed within a collet on a lathe stage (LaserTurn1, Aerotech, Inc., Pennsylvania, United States) during the notching procedure. The materials used for the hinge design included PC and PEI, with respective Young's moduli of 2400 MPa and 3500 MPa, and admissible elastic strains of 5% and 6%.

To encapsulate the braid reinforcement and reduce thrombogenesis, the catheter was covered with two layers of polyethylene heat shrink tubing (6 μm thick, 103-0042, Nordson MEDICAL, New Hampshire, United States). Prior to activating the heat shrink tubing, a short segment of low shore hardness platinum-cured silicone tubing (SFM3-2050, Polymer Systems Technology Ltd, High Wycombe, United Kingdom) was attached to the distal end, creating an atraumatic tip.

For the coating process, a urethane-based primer (Primer 5-017, Coatings2Go, Massachusetts, United States) and a water-based hydrophilic coating (Patented Hydrophilic Coating 8-3C, Coatings2Go, Massachusetts, United States) were used. The catheter samples were first wiped with isopropanol and then placed in an oxygen plasma oven (Atto, Diener electronic GmbH & Co. KG, Ebhausen, Germany) for 5 minutes to clean and activate the surface, improving adhesion characteristics. The plasma-cleaned assembly was then manually dip-coated in the urethane-based primer to promote adhesion to the subsequent hydrophilic topcoat. After drying for 5 minutes at 80°C, the assembly was manually dip-coated in the hydrophilic coating. Since the primary goal is to reduce friction between the catheter and the vascular wall, the evenness of the coating thickness is not crucial. Nonetheless, the dip coating process was performed at a consistent speed to achieve as uniform coverage as possible. The coated catheter was then dried in the draw tower's 3-zone tube furnace at 80°C for 8 hours to ensure a durable coating. Finally, two passive negative markers, doped with iron microparticles, were added to the distal and proximal ends of the deflectable tip to enhance MR visibility.

**Notes S4: Post-processing of active tracking catheter**

A Tiger-shaped tip was achieved using a 3D-printed mold with a specific slot designed for the catheter tip. The catheter tip was placed in this mold and left for several days to fully relax internal stresses, ensuring that the tip retained its Tiger shape after removal. Following this, a resonance coil was attached to the tip of the catheter. This coil was then connected to a coaxial wire embedded within the catheter shaft to facilitate active tracking during MRI procedures. To join the COCe tip with the PC shaft, a PTFE shrinking tube was used. The PTFE tube was heated to securely bond the COCe tip to the PC shaft, ensuring a robust and reliable connection.

**Notes S5: Methodology for flexibility characterization (Experiment 1-1, Experiment 1-2 and Experiment 1-3)**

The measurement of flexural rigidity (*EI*), which represents the stiffness of a material when subjected to bending, is conducted to assess the force required to bend a catheter shaft with a specific extruded length. This test utilizes the principles of cantilever beam theory to calculate the flexural rigidity as $EI\ = \frac{FL^3}{3\delta}$, where $L$ is the length of the catheter extension and δ is the displacement resulting from the applied force F at the distal end of the catheter (Figure S6B). The flexural rigidity is determined by the product of the elastic modulus (*E*) and the moment of inertia of the catheter section (*I*), and it is essential to ensure that the catheter deflection does not exceed the material's elastic limit to comply with the assumptions of the cantilever beam theory.

Experiment 1-1 focuses on evaluating the flexural rigidity of the catheter shaft. A catheter sample with an extruded length of *L* = 20 mm is clamped in a pin vise, and a push rod positioned above the free end applies a force that induces a small deflection δ of 5mm. The opposite end of the push rod is connected to a force/torque sensor (ATI F/T Sensor Nano43, ATI Industrial Automation, North Carolina, United States), which measures the force required to push the sample. To achieve precise positioning, the push rod/sensor assembly is mounted on a linear stage (M4004M, Parker, Pennsylvania, United States) driven by a servo motor (Dynamixel MX-106T, Robosavvy, London, United Kingdom). The push rod moves at a constant speed until the catheter reaches the desired deflection position and remains in that position for 12 seconds before returning to its initial location at the same speed. Thirteen different types of catheters are examined, with each type tested 5 times.

In Experiment 1-2, the impact of moisture absorption on the flexural rigidity is investigated. The catheter samples are immersed in a simulated intravascular environment with water at 37.4 °C for 30 min, and Experiment 1-1 is repeated within the circulating water tank at constant temperature. Each catheter type undergoes four tests to assess the potential influence of moisture absorption on stiffness and its implications for in-vivo catheter performance . Thirteen different types of catheters are examined, with each type tested 4 times.

Experiment 1-3 focuses on the relaxation effect exhibited by polymers, wherein the bending stiffness decreases over time [71]. In this experiment, the push rod holds the catheter in place for 30 minutes while recording the change in force during this period. Each type of catheter undergoes this test once to characterize the relaxation property.

**Notes S6: Methodology for pushability characterization (Experiment 2)**

The pushability of a catheter, which represents its ability to transmit force in the axial direction, is an important characteristic. A higher pushability allows for smoother advancement and puncture within the vascular network. This experiment aims to assess the pushability of various catheter specifications by measuring their axial stiffness (*EA*). The magnitude of pushability is often determined by the product of the material's elastic modulus (*E*) and the cross-sectional area of the catheter shaft (*A*), which is calculated using the equation $EA = \frac{PL}{\delta}$. Here, $L$ denotes the length of the fixed catheter sample, $P$ represents the force required to push one end to a specified displacement δ, and the other end is secured.

Experiment 2 focuses on examining the pushability of the catheter shafts. As depicted in Figure S7, B and C, a catheter shaft sample is clamped in place using two pin vises positioned *L* = 120 mm apart. One pin vise is connected to a force/torque sensor (ATI F/T Sensor Nano43, ATI Industrial Automation, North Carolina, United States) to measure the reaction force P necessary to push the catheter shaft sample a displacement δ = 5 mm. The assembly of the pin vise and force/torque sensor is mounted on a linear stage (M4004M, Parker, Pennsylvania, United States) coupled with a servo motor (Dynamixel MX-106T, Robosavvy, London, United Kingdom) to achieve the desired displacement. Each catheter shaft sample undergoes testing at least 3 times to obtain reliable results.

**Notes S7: Methodology for torqueability characterization (Experiment 3-1 and Experiment 3-2)**

Torqueability refers to a catheter's ability to transfer torque from the handle to the distal end. A high torquability is desired for effective manipulation at the handle end. The torsional rigidity (*GJ*), calculated as the product of the shear modulus of stiffness (*G*) and the moment of inertia of the cross-sectional area (*J*), is commonly used to quantify torquability. To apply beam theory equations and calculate the *GJ* of the catheter, it is assumed that strain is linear within the range of applied torsional angles. The relationship for *GJ* is expressed as $GJ = \frac{TL}{\varphi}$, where *L* represents the fixed length of the catheter and *T* denotes the torque required to rotate the catheter by the angle φ in radians.

Experiment 3-1 involves clamping the catheter shaft sample between two pin vises positioned at a distance of L = 120mm, as illustrated in Figure S8B. One of the pin vises is connected to a servo motor (Dynamixel MX-106T, Robosavvy, London, United Kingdom) to apply torque to the sample, resulting in a twist angle (φ = 2π). The other pin vise is attached to a static

force/torque sensor (ATI F/T Sensor Nano43, ATI Industrial Automation, North Carolina, United States) to measure the torque *T*. Each catheter shaft sample undergoes a minimum of 5 tests, and torque is measured at the 12th second after twisting the catheter shaft sample about its axis.

Experiment 3-2 utilizes a setup inspired by Yildirim et al. [70], as depicted in Figure S9. The aim is to measure the torque response by placing the catheter shaft samples in a low-friction PTFE vessel phantom that mimics the trajectory of a transfemoral catheter across the aortic arch and descending thoracic aorta. The proximal end of the catheter shaft is firmly clamped to a pin vise connected to a servo motor (Dynamixel MX-106T, Robosavvy, London, United Kingdom). Rotation of the motor causes the catheter to twist, simulating the motion of a surgeon rotating the catheter's proximal handle/hub during an intervention. A marker is attached to the distal end of the sample to track its corresponding twist using a high-sensitivity USB 3.0 CMOS camera (DCC3240M, Thorlabs GmbH, Bergkirchen, Germany). The experiment is repeated a minimum of 4 times for each catheter shaft sample to ensure reliable data collection.

**Notes S8: Methodology for steerability characterization (Experiment 4-1 and Experiment 4-2)**

Steerability refers to the ability of a catheter to deflect its distal end. This section focuses on investigating the steerability of the integrated catheter-handle assembly, specifically examining repeatability and the torque required to turn the knob, which demonstrates its steering performance.

Experiment 4-1: An experimental setup is devised to evaluate the steerability of the catheter's distal end, assess its workspace, and measure positioning accuracy (Figure S10). The experiment begins by securing the distal end of the catheter using a pin vice, while the proximal handle is secured in place. To conduct the experiments, one of the handle's external covers is removed, and a servo motor (Dynamixel MX-106T, Robosavvy, London, United Kingdom) is connected in-line with the knob. A force/torque sensor (ATI F/T Sensor Mini40, ATI Industrial Automation, North Carolina, United States) and a coupling, connecting the rotating force/torque sensor (driving mechanism) to the knob (driven mechanism), are placed between the knob and the motor. The coupling consists of an internal gear with the same diameter and modulus as the knob. The force/torque sensor measures the torque required to turn the knob.

The knob, driven by the motor, undergoes a series of rotations. Firstly, it is turned 90° in one direction, causing the catheter tip to bend (Stroke I - Forward Steering). Then, the motor is reversed to return the knob and catheter tip to their initial position (Stroke II - Backward Steering). Next, the knob is rotated an additional 90° in the opposite direction, causing the catheter tip to bend in the other direction (Stroke III - Forward Steering). Finally, the knob is turned back to its initial home position (Stroke IV - Backward Steering). Each 90° rotation consists of eight equal steps, with the motor rotating in 11.25° increments. The deflection of the catheter tip is recorded using a high-sensitivity USB 3.0 CMOS camera (DCC3240M, Thorlabs GmbH, Bergkirchen, Germany) positioned perpendicular to the plane of motion. Additionally, four dark markers are attached equidistantly along the catheter tip to facilitate tracking and depict its shape using computer vision methods. The degrees of freedom for up/down and left/right steering of the catheter tip are tested separately.
Experiment 4-2: During catheter navigation in the vascular network, passive bending occurs inevitably. It is crucial to evaluate the steering performance after passive bending. In this experiment (Figure S11), a fixed pulley is placed in the middle of the catheter shaft, resulting in a 90° bend of the catheter. After adjusting the knob to straighten the catheter tip and compensate for passive tip deflections, the same experimental protocol described in Experiment 4-1 is repeated in this bent configuration.

**Notes S9: Methodology for helical compensation characterization (Experiment 5)**
Conventional pull-wire driven catheters exhibit three undesirable phenomena during interventions: passive tip deflection, muscling, and curve alignment. This section focuses on investigating the suppression of passive tip deflections using helically routed pull-wires.
Experiment 5: The objective of this experiment is to evaluate the effectiveness of helically routed pull-wires in compensating for passive deflections. To achieve this, the catheter shaft is passively bent around a vee-grooved pulley, serving as a pivot point (RS Part No. 352-0664, RS Components, Corby, United Kingdom) as depicted in Figure S12. The experiment begins by mounting the integrated catheter handle assembly onto a sliding carriage (NW-02-17, Igus, Northampton, United Kingdom). The carriage, in turn, is mounted on a linear rail (NS-01-17-300, Igus, Northampton, United Kingdom), which is fixed to a 3D printed rotating beam. This setup allows the catheter handle assembly to slide freely along the rail while rotating around the pivot point. At one end of the beam, there is an additively manufactured spur gear connected to a passive rotary stage (PR01/M, Thorlabs GmbH, Bergkirchen, Germany). Positioned on top of this gear is the pivoting pulley, which guides and maintains alignment of

the catheter shaft within its grooves during the rotation of the catheter-handle assembly. The spur gear is driven by another gear directly connected to a servo motor (Dynamixel MX-106T, Robosavvy, London, United Kingdom). At the distal end, the flexible tip of the catheter is clamped in place by a pin vice, securing it from its proximal end.

Driven by the motor, the catheter shaft on the beam rotates around the pulley, forming a 90° angle as indicated by the yellow arrow. Subsequently, the beam returns to its initial home position, concluding the experiment. Concurrently, the movement of the catheter tip is recorded by tracking a marker positioned at its distal-most point. The motion of the tip is captured using a high-sensitivity USB 3.0 CMOS camera (DCC3240M, Thorlabs GmbH, Bergkirchen, Germany). In this experiment, five catheter-handle assemblies (two helical and three parallel lumina) are tested. Each catheter assembly undergoes testing 4 times.

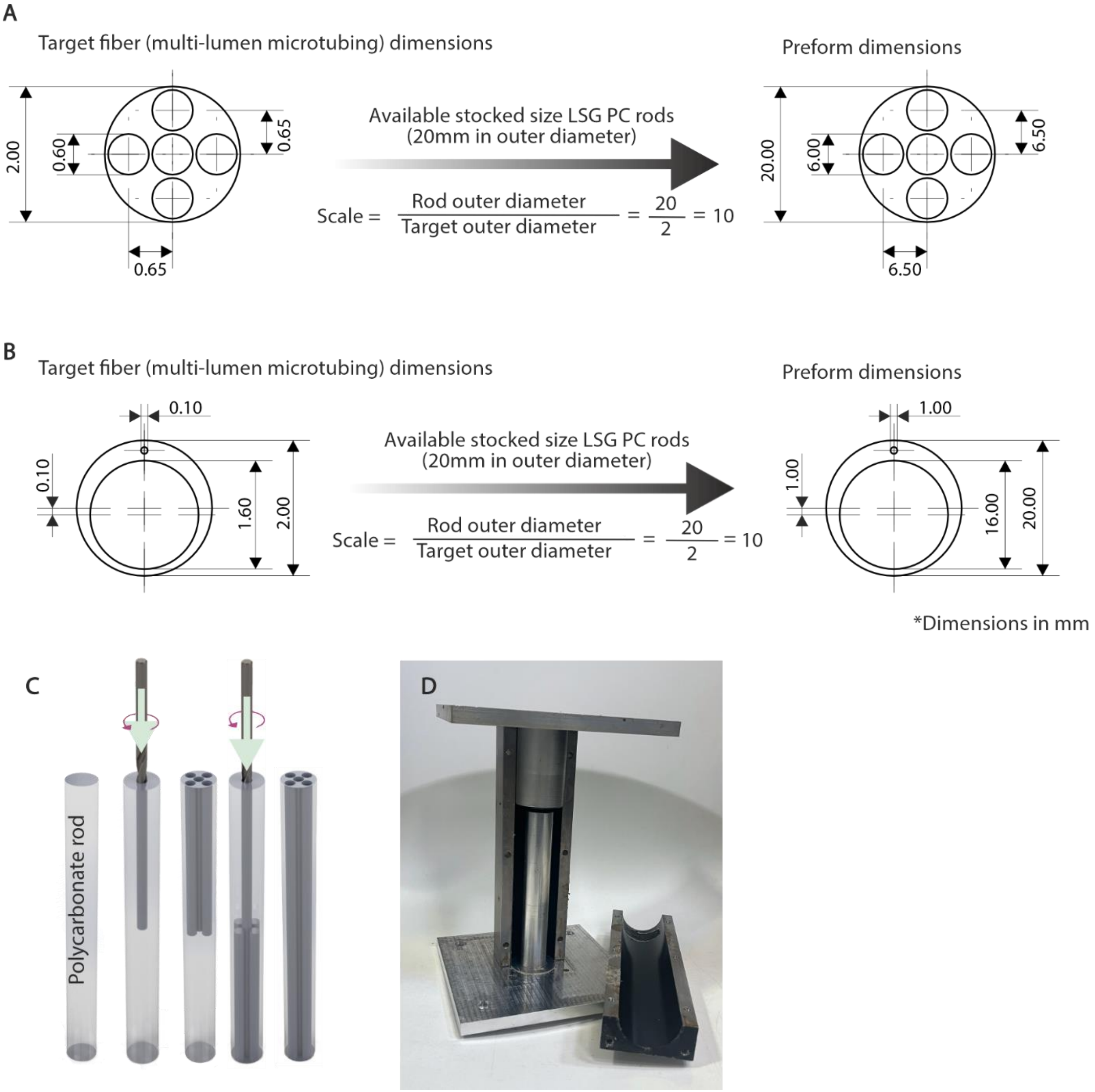


**Figure S1. Designs and fabrication of catheter and preform.** (**A**) Calculation of the fiber-to-preform ratio for the steerable catheter. (**B**) Calculation of the fiber-to-preform ratio for the active tracking catheter. (**C**) CAD representation of the preform fabrication process for the steerable catheter: polymeric rod peck drilling performed form both ends. (**D**) Photograph of the mould used to fabricate the COCe preform.

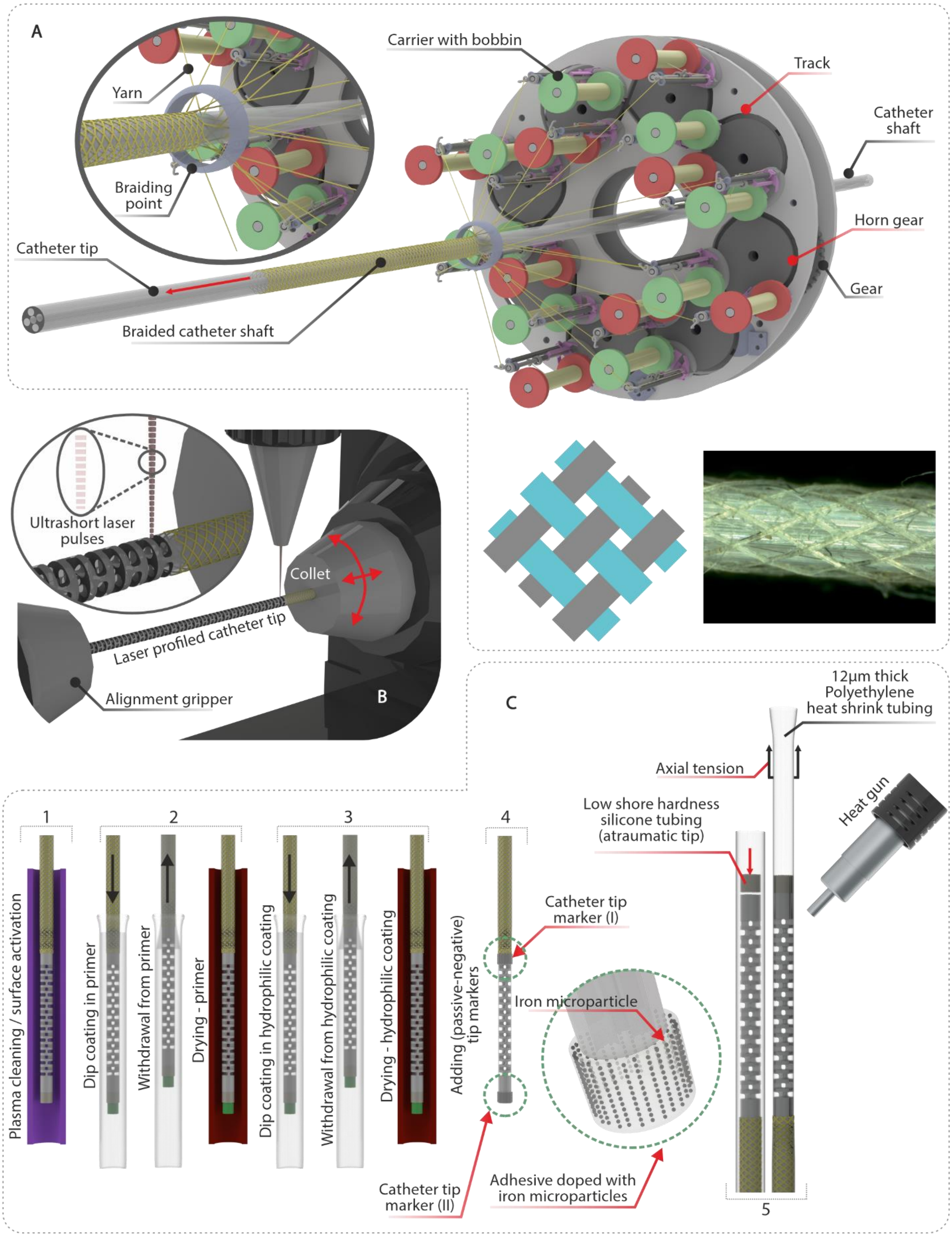


**Figure S2. Post-process procedures for steerable catheter.** (**A**) CAD representation of the Maypole braider used to overbraid the uncovered thermally drawn multi-lumen tubing. (Note: The distal end of the tubing remains unbraid to facilitate subsequent laser profiling.) Close-up view of the braiding point where the yarns intersect. Diamond (1/1) braiding patterns

achievable with the Maypole braider. Optical images depicting thermally drawn polycarbonate fibers (multi-lumen tubing) that have undergone overbraiding. (**B**) CAD representation of the laser profiling setup. Close-up view of the ultrashort laser pulses used for non-thermal ablation of the polymeric substrate. (**C**) CAD illustration of the coating heat shrink covering procedures. (1) Plasma cleaning and surface activation of the catheter assembly by wiping the catheter samples with isopropanol and placing them in an oxygen plasma oven (Atto, Diener electronic GmbH & Co. KG, Ebhausen, Germany) for 5 minutes. (2) A blocker (indicated in green) is added at the distal end to prevent coating material from entering and blocking the lumina. Dip coating the assembly in primer (Primer 5-017, Coatings2Go, Massachusetts, United States). After removal from the primer, Drying the primer-coated assembly for 5 minutes at 80°C. (3) Dip coating the assembly in a hydrophilic coating. Removal from the hydrophilic coating and dry the hydrophilic-coated assembly for 8 hours at 80°C. (4) Addition of passive negative tip markers for visualization under MRI. (5) Apply heat shrink tubing to cover the overbraided and laser-cut multi-lumen tubing (fiber).

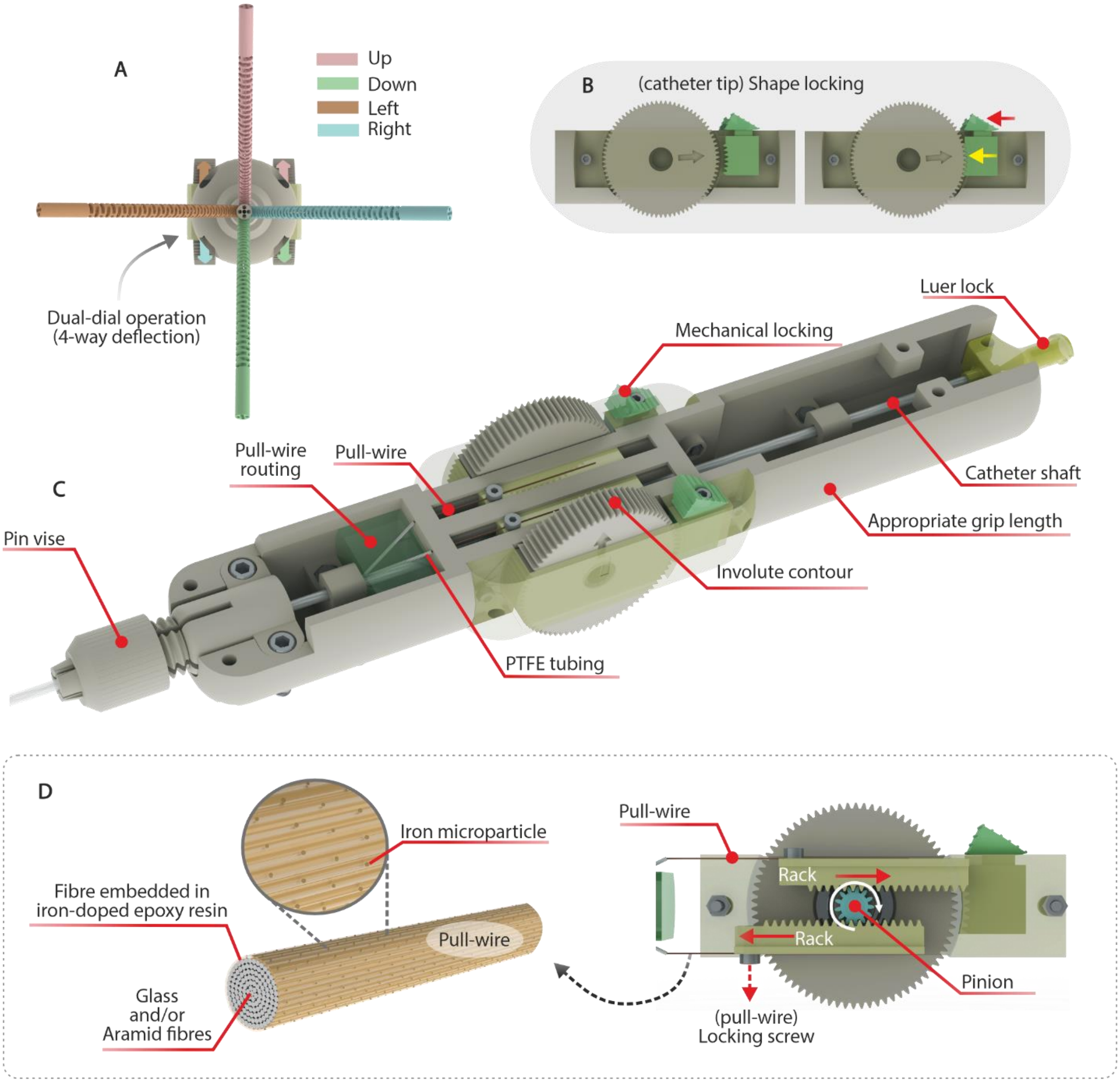


**Figure S3. CAD of the steerable catheter handle integration.** (**A**) The handle allows for remote control of the tip's 4-way deflection using dual dial knobs, with different colors representing bending directions. (**B**) Shape locking mechanism. (**C**) Isometric view shows the catheter handle assembly without the top cover, featuring a dual rack and pinion mechanism and a shape locking mechanism. (**D**) The handle incorporates MR visible pull-wires for catheter visualization, as depicted in the zoomed-in view of the doped iron microparticles.

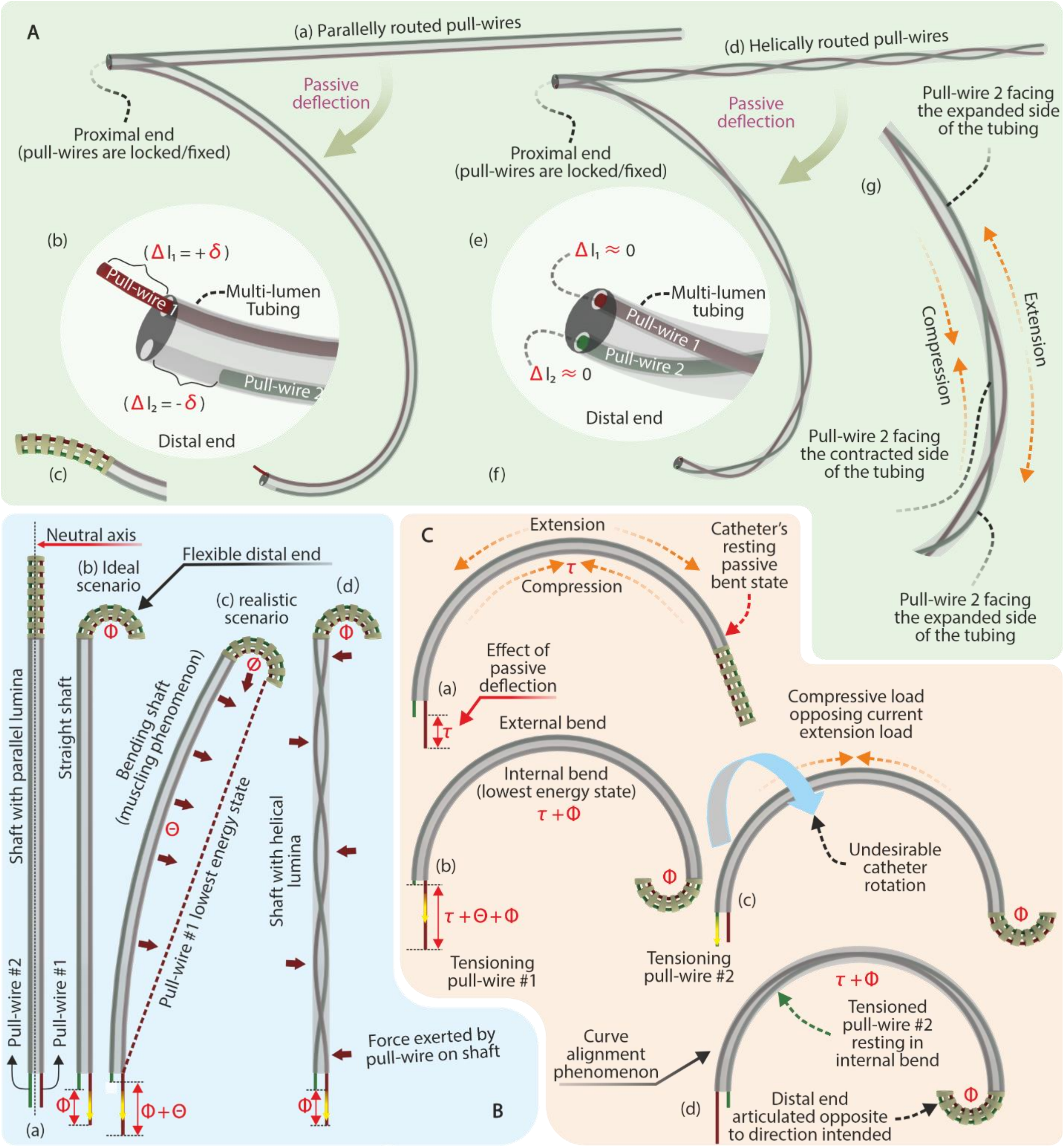


**Figure S4. Three phenomena observed during the manipulation of flexible instruments in complex anatomies.** (**A**) Passive Tip Deflection Phenomenon: (a,b) Uncontrolled lengthening (+δ) and shortening (-δ) of pull-wires due to passive deflection of a flexible shaft with parallel pull-wire routing. (c) Undesirable bending of the flexible distal end of the catheter, where the pull-wires are anchored. (d,e,f) Pull-wires routed in helical lumina experience counterbalanced extension and compression forces, resulting in minimal changes in length ($\delta \approx 0$). (**B**) Muscling phenomenon: (h) Neutral axis of the catheter. (i) Ideal catheter steering, where pull-wire tension deflects only the flexible distal end by φ. (j) Realistic catheter steering, where tensioning of the pull-wire deflects the shaft by θ, resulting in a total

catheter deflection of φ+θ. (k) Counterbalancing of opposing compressive and tensile forces minimizes the muscling phenomenon. (**C**) Curve alignment phenomenon: (l) Catheter in a passive bent state with a radius angle of curvature τ. (m) Tensioning of the pull-wire along the internal bend leads to cumulative bending of τ+θ+φ. (n) Subsequent tensioning of the opposing pull-wire applies a compressive load opposing the current extension loads for bending the catheter in its resting state. (o) Undesirable shaft rotation as tensioned pull-wires attempt to settle in the internal bend.

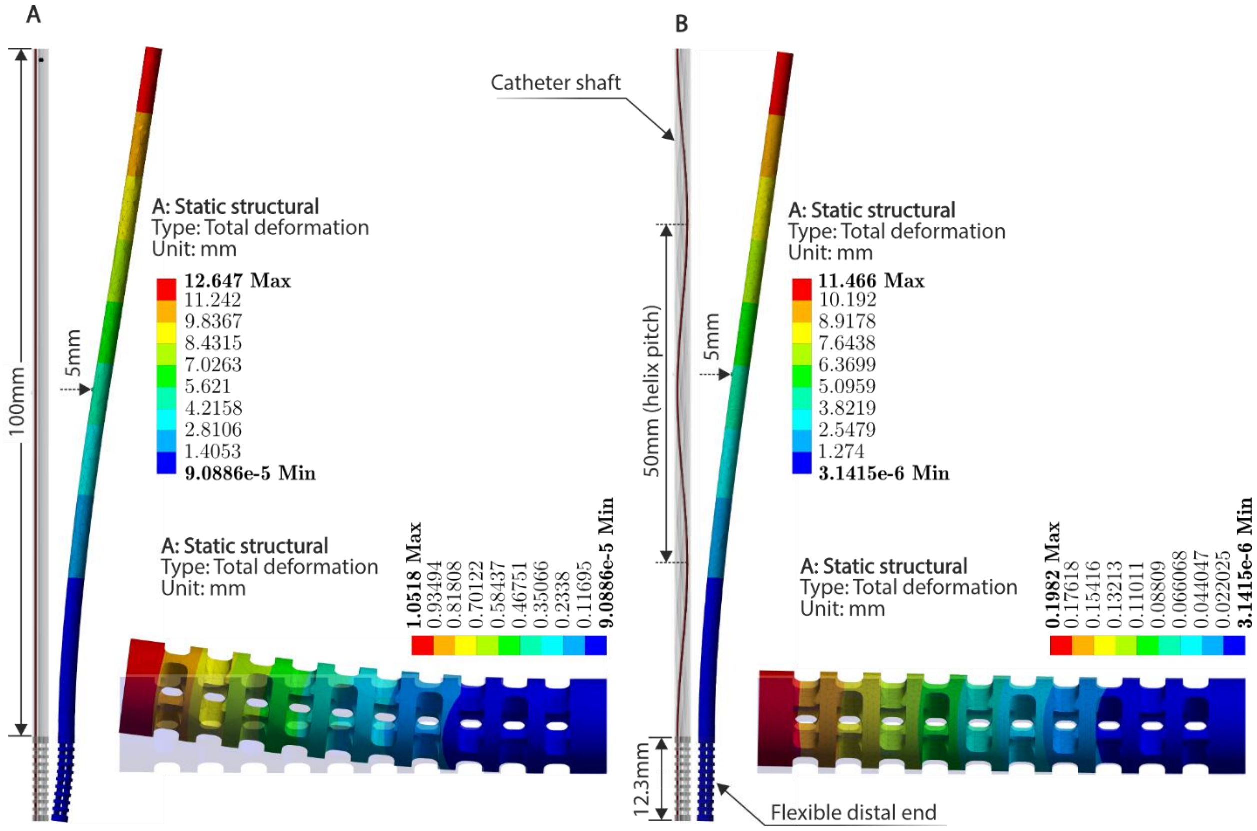


**Figure S5. FEM static structural analysis.** (**A**) Steerable catheter with a flexible distal end and a parallelly routed pull-wire, anchored at both ends of the catheter. The catheter shaft undergoes a passive deflection of 5mm midway to simulate passive deflection. Total deformation contours of the flexible distal end for the parallel case. (**B**) The catheter with helically routed pull-wire is subjected to the same boundary conditions and loads, with the proximal end of the flexible distal end fixed. The total deformation analysis of the flexible distal end for the helical case is shown. The simulation settings and parameters include the catheter material: PC, pull-wire material: stainless steel, and a coefficient of friction between the pull-wire and catheter of 0.2. The vertical colour scale bar indicates the displacement of the catheter shaft from its original position under an applied displacement of 5 mm at the middle; the horizontal colour scale bar indicates the uncontrolled displacement of the catheter tip from its original position.

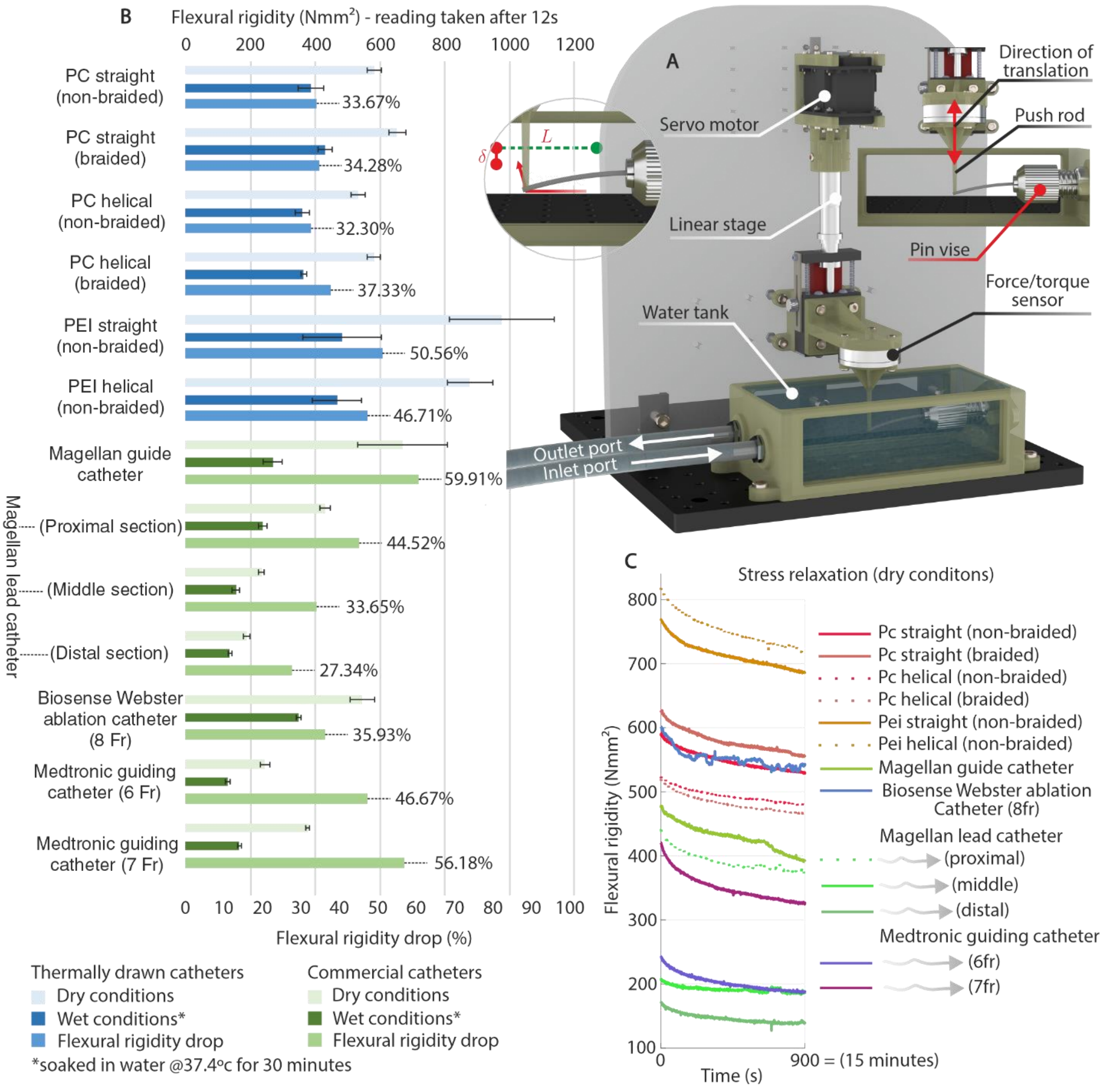


**Figure S6. Experimental setup and results for Experiment 1-1, Experiment 1-2 and Experiment 1-3.** (**A**) Results of flexural rigidity under dry condition (Experiment 1-1; n = 5 for each category) and after water immersion (Experiment 1-2; n = 4 for each category). (**B**) CAD representations of the experimental setup. (Left) Close-up view illustrating the maximum deflection (δ) and the length of the sample (L). (Right) Close-up view showing the direction of translation of the push rod and force/torque assembly. (**C**) Results of stress relaxation in Experiment 1-3.

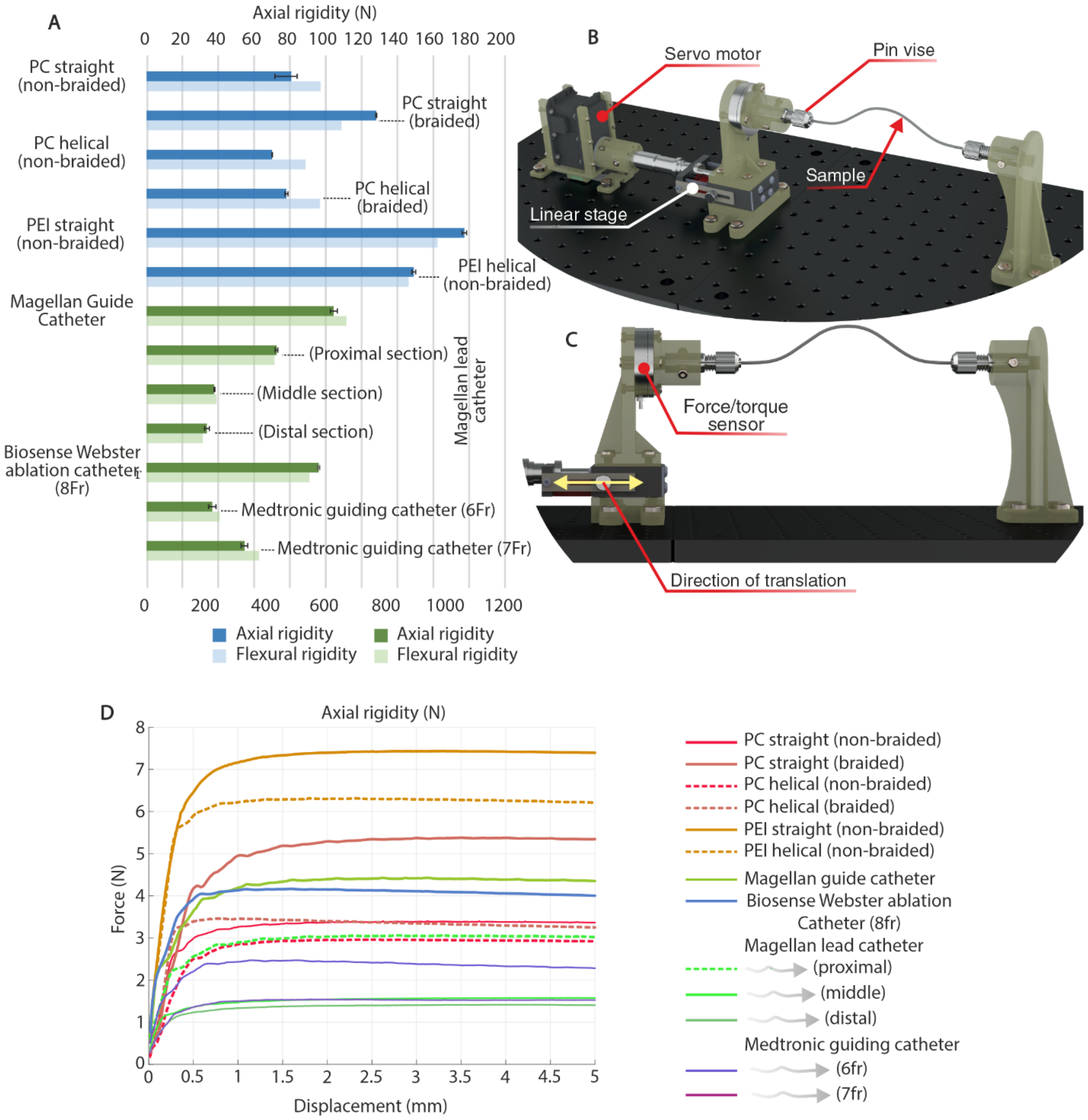


**Figure S7. Experimental setup and results for Experiment 2.** (**A**) Experimental results of the axial rigidity (Experiment 2; n = 4 for distal section of Magellan lead catheter, Medtronic guide catheter, PC helical braided catheter and PC straight unbraided catheter, n = 3 for all others) comparing with flexural rigidity (Experiment 1-1). (**B**, **C**) CAD representations of the experimental setup. (**D**) Graph showing the relationship between force and displacement.

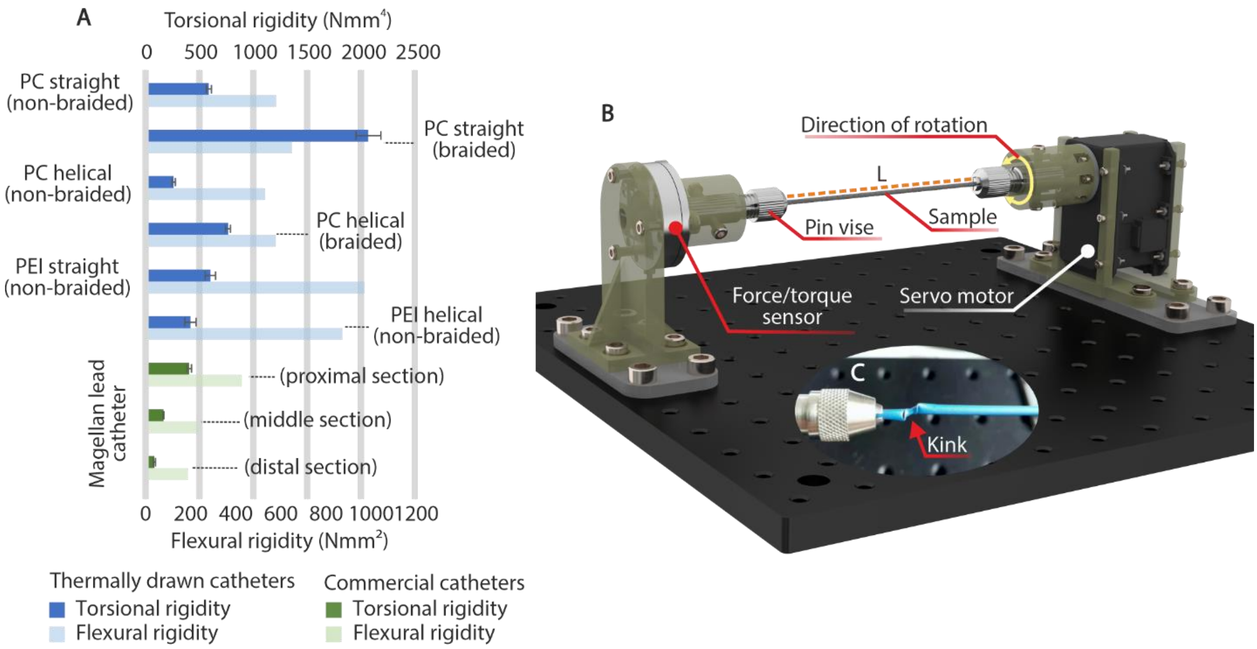


**Figure S8. Experimental setup and results for Experiment 3-1.** (**A**) Experimental results of the torsional rigidity (Experiment 3-1; n = 5 for each category) comparing with flexural rigidity (Experiment 1-1). (**B**) CAD representations of the experimental setup. (**C**) Image of a kinked catheter sample.

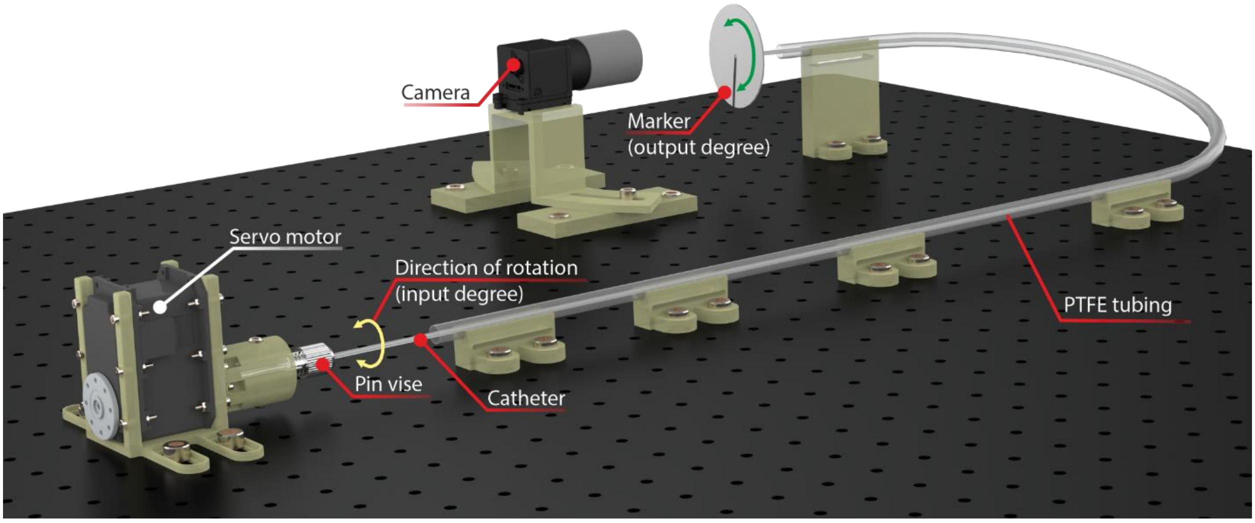


**Figure S9. CAD representations of the experimental setup for Experiment 3-2.**

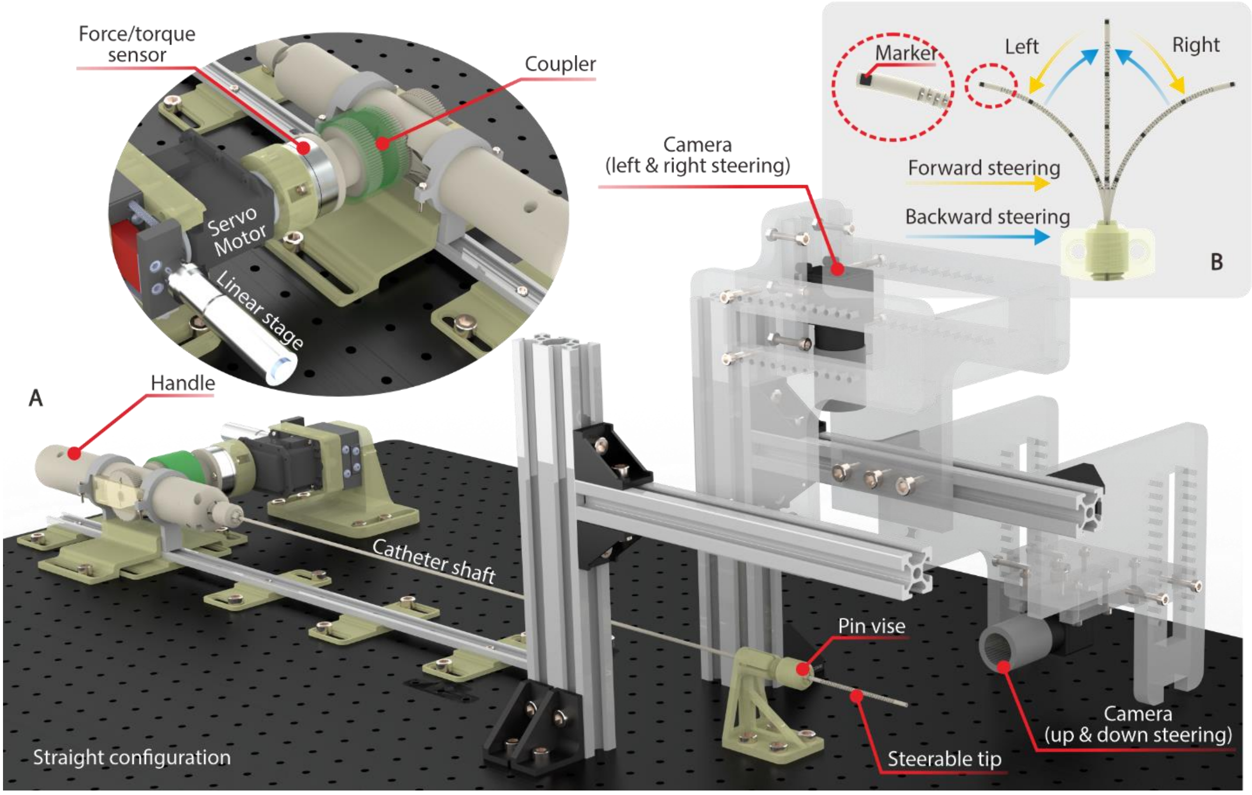


**Figure S10. Setup for Experiment 4-1: catheter steering in a bent configuration.** (**A**) CAD representations of the experimental setup. (**B**) Definitions of forward and backward steering and marker added to the distal end for tracking purposes.

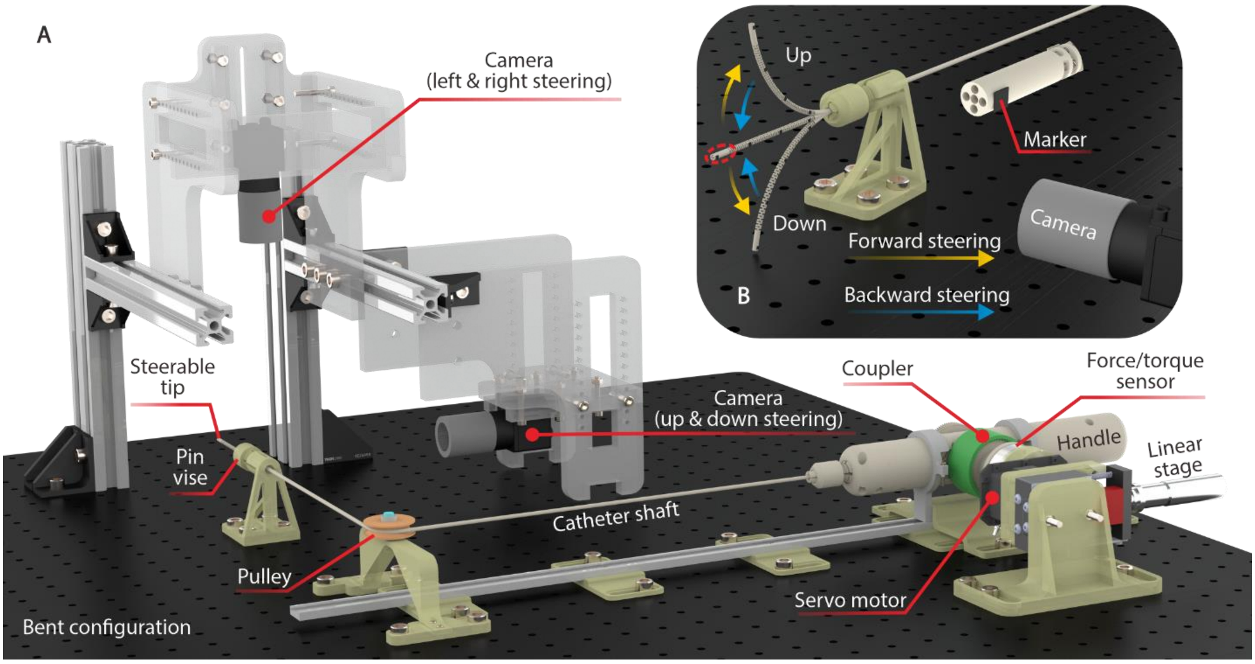


**Figure S11. Setup for Experiment 4-2: catheter steering in a bent configuration.** (**A**) CAD representations of the experimental setup. (**B**) Definitions of forward and backward steering and marker added to the distal end for tracking purposes.

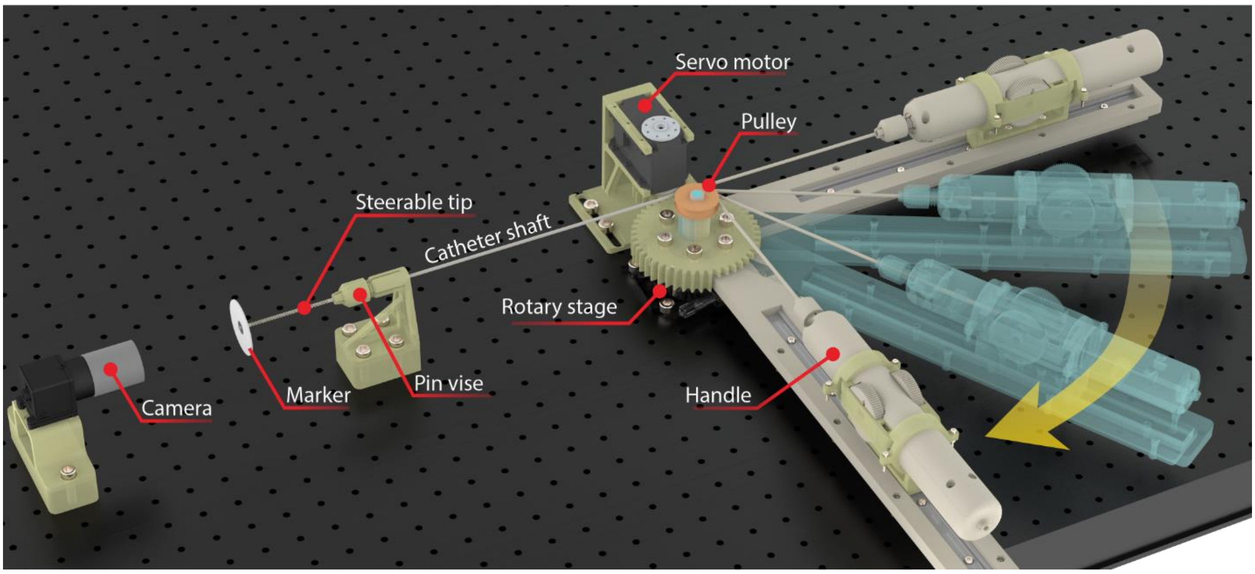


**Figure S12. CAD representations of the experimental setup for Experiment 5.**

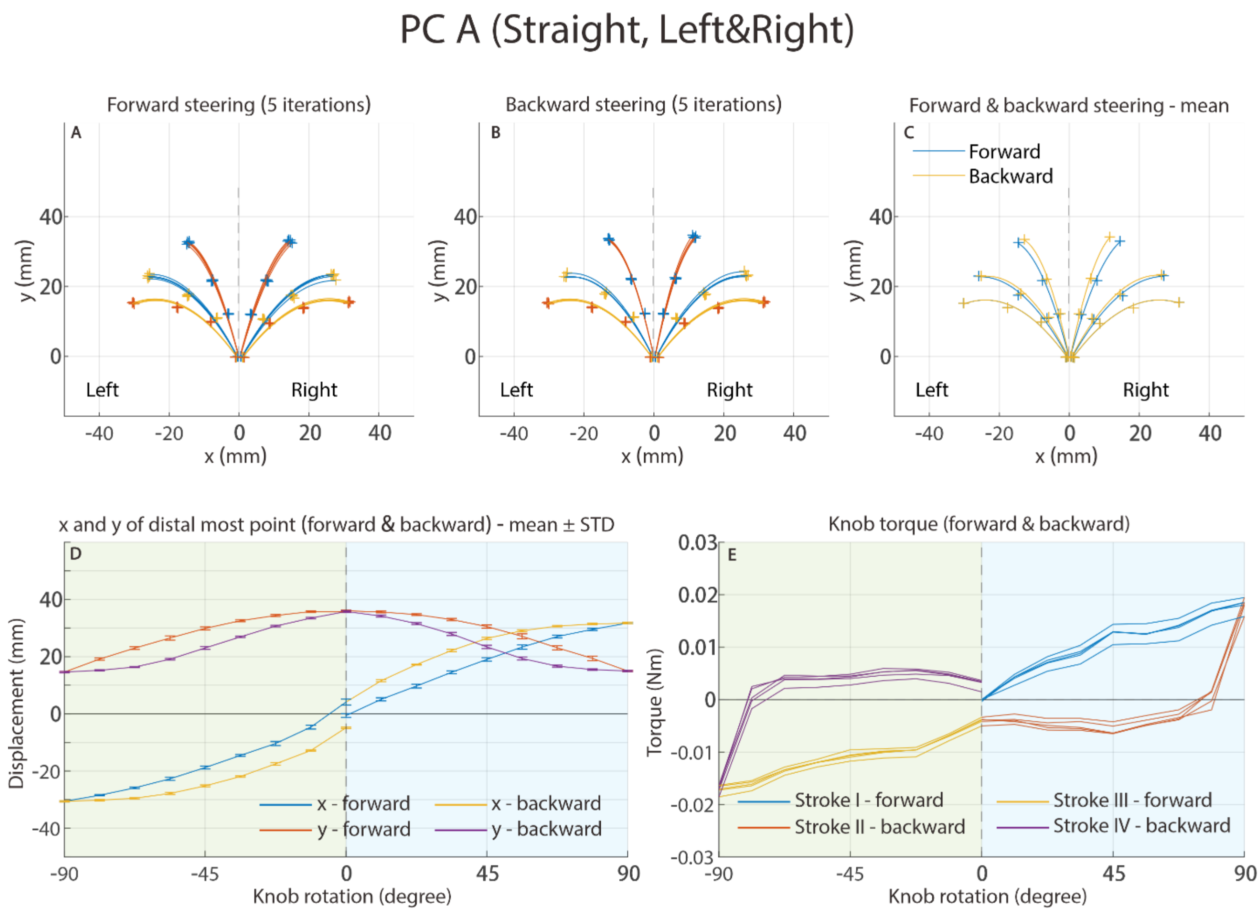


**Figure S13. The results of Experiments 4-1 show the left and right tip motion of PC A catheter in a straight configuration.** (**A**) The geometry of the catheter for each trial when the catheter is steered forward (Stroke I and Stroke III). (**B**) The geometry of the catheter for each trial when the catheter is steered backward (Stroke II and Stroke IV). (**C**) The catheter profiles during the steering at each step (with a step length of 22.5°) are plotted by averaging the catheter positions over multiple trials with different knob angular positions. Backlash is indicated by the difference in catheter positions for the same knob angle position during forward and backward strokes. To aid visualization, only shape diagrams for knob rotations of ± 22.5°, ± 56.25°, and ± 90°are shown. (**D**) displays the average coordinates (with standard deviation) of the catheter tip positions in the x and y directions throughout the experiment. (**E**) displays the torque applied to the knob during the experimental procedure, along with its standard deviation.

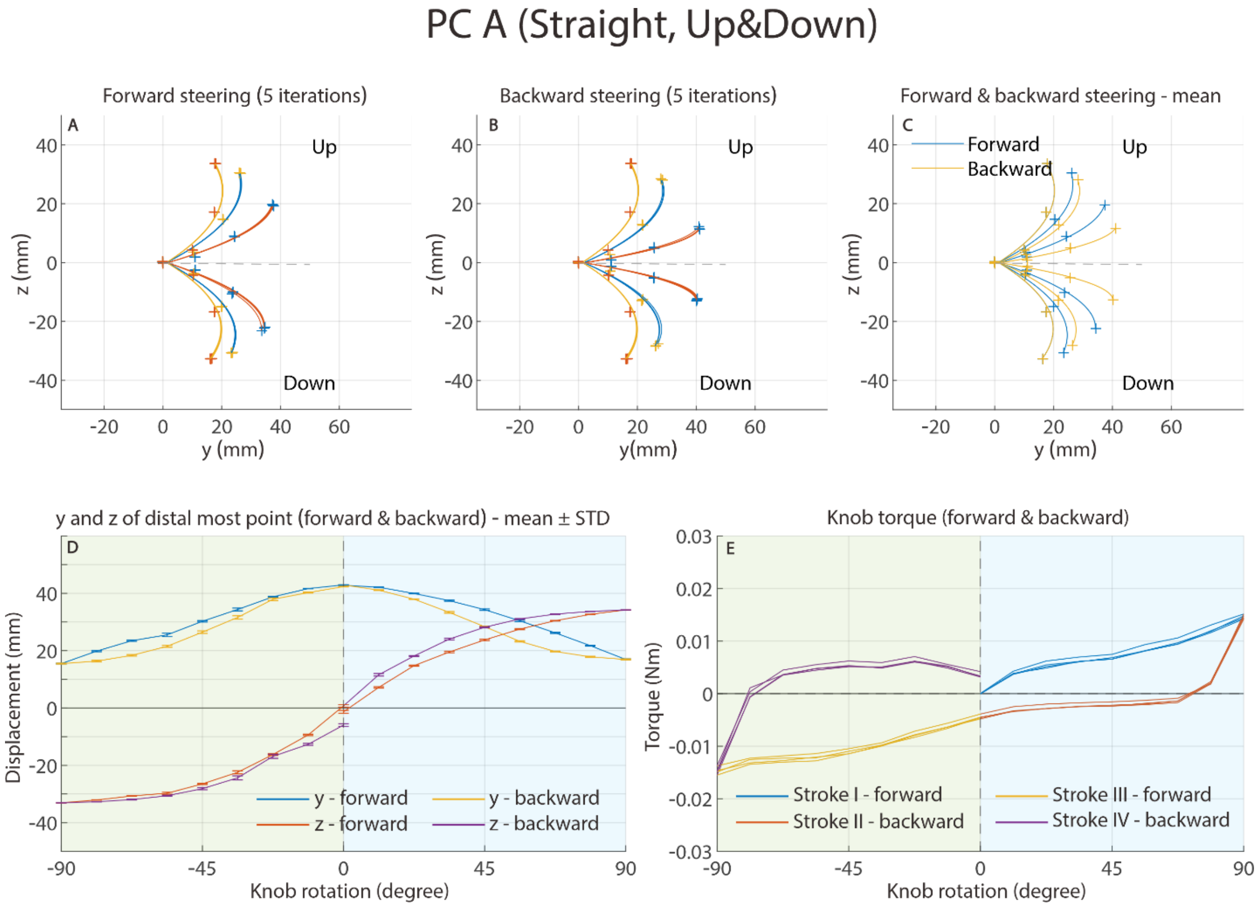


**Figure S14. The results of Experiments 4-1 show the up and down tip motion of PC A catheter in a straight configuration.** (**A**) The geometry of the catheter for each trial when the catheter is steered forward (Stroke I and Stroke III). (**B**) The geometry of the catheter for each trial when the catheter is steered backward (Stroke II and Stroke IV). (**C**) The catheter profiles during the steering at each step (with a step length of 22.5°) are plotted by averaging the catheter positions over multiple trials with different knob angular positions. Backlash is indicated by the difference in catheter positions for the same knob angle position during forward and backward strokes. To aid visualization, only shape diagrams for knob rotations of ± 22.5°, ± 56.25°, and ± 90°are shown. (**D**) displays the average coordinates (with standard deviation) of the catheter tip positions in the x and y directions throughout the experiment. (**E**) displays the torque applied to the knob during the experimental procedure, along with its standard deviation.

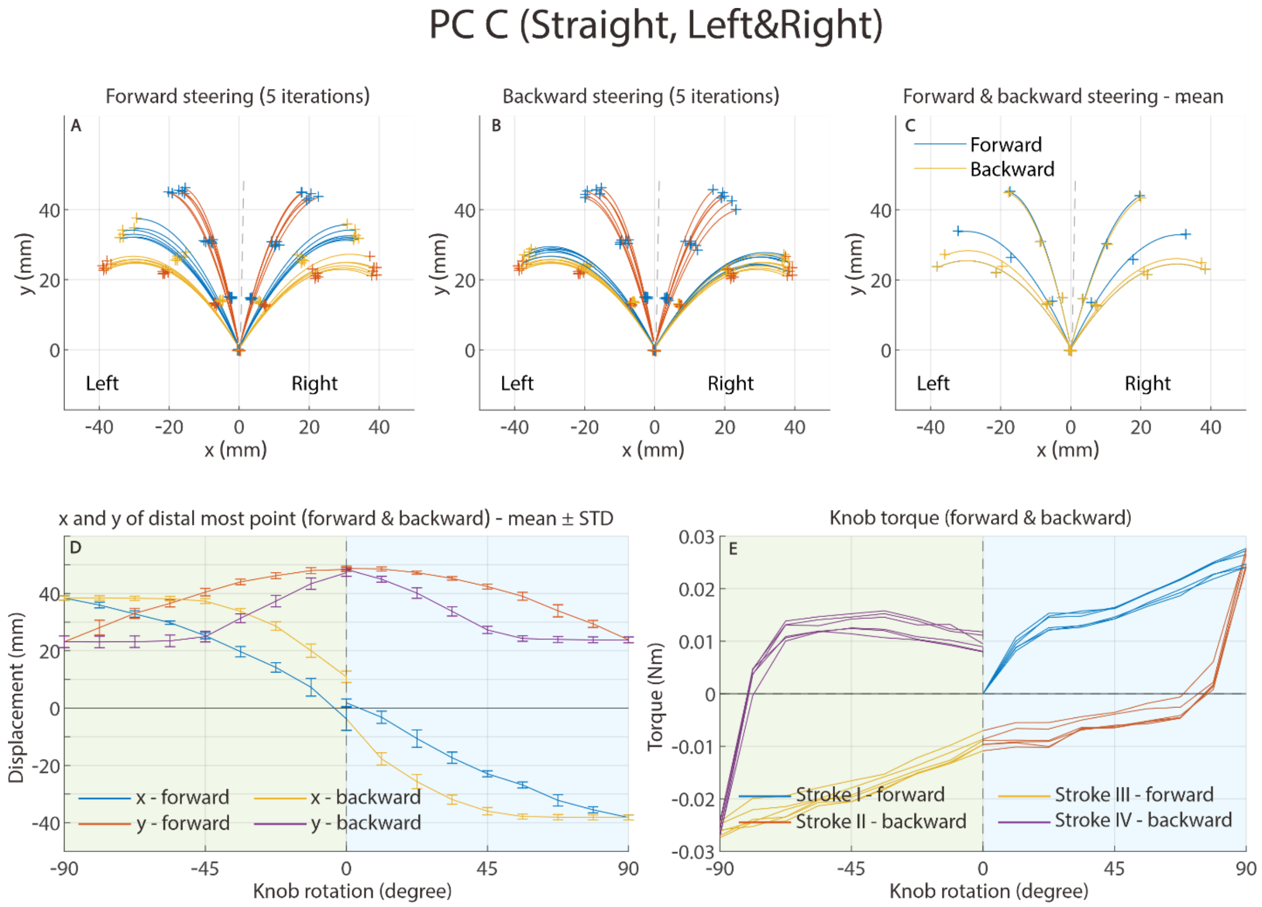


**Figure S15. The results of Experiments 4-1 show the left and right tip motion of PC C catheter in a straight configuration.** (**A**) The geometry of the catheter for each trial when the catheter is steered forward (Stroke I and Stroke III). (**B**) The geometry of the catheter for each trial when the catheter is steered backward (Stroke II and Stroke IV). (**C**) The catheter profiles during the steering at each step (with a step length of 22.5°) are plotted by averaging the catheter positions over multiple trials with different knob angular positions. Backlash is indicated by the difference in catheter positions for the same knob angle position during forward and backward strokes. To aid visualization, only shape diagrams for knob rotations of ± 22.5°, ± 56.25°, and ± 90°are shown. (**D**) displays the average coordinates (with standard deviation) of the catheter tip positions in the x and y directions throughout the experiment. (**E**) displays the torque applied to the knob during the experimental procedure, along with its standard deviation.

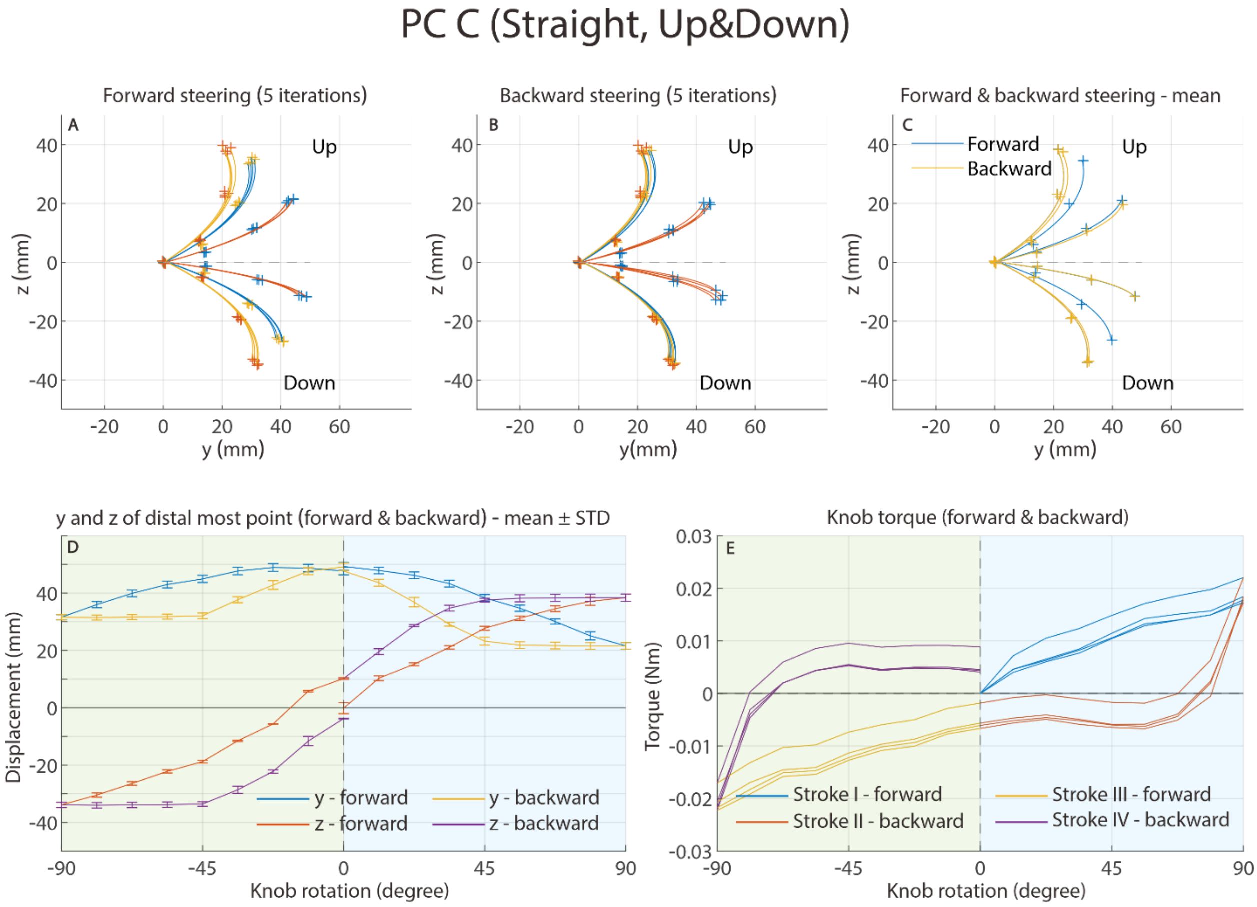


**Figure S16. The results of Experiments 4-1 show the up and down tip motion of PC C catheter in a straight configuration.** (**A**) The geometry of the catheter for each trial when the catheter is steered forward (Stroke I and Stroke III). (**B**) The geometry of the catheter for each trial when the catheter is steered backward (Stroke II and Stroke IV). (**C**) The catheter profiles during the steering at each step (with a step length of 22.5°) are plotted by averaging the catheter positions over multiple trials with different knob angular positions. Backlash is indicated by the difference in catheter positions for the same knob angle position during forward and backward strokes. To aid visualization, only shape diagrams for knob rotations of ± 22.5°, ± 56.25°, and ± 90°are shown. (**D**) displays the average coordinates (with standard deviation) of the catheter tip positions in the x and y directions throughout the experiment. (**E**) displays the torque applied to the knob during the experimental procedure, along with its standard deviation.

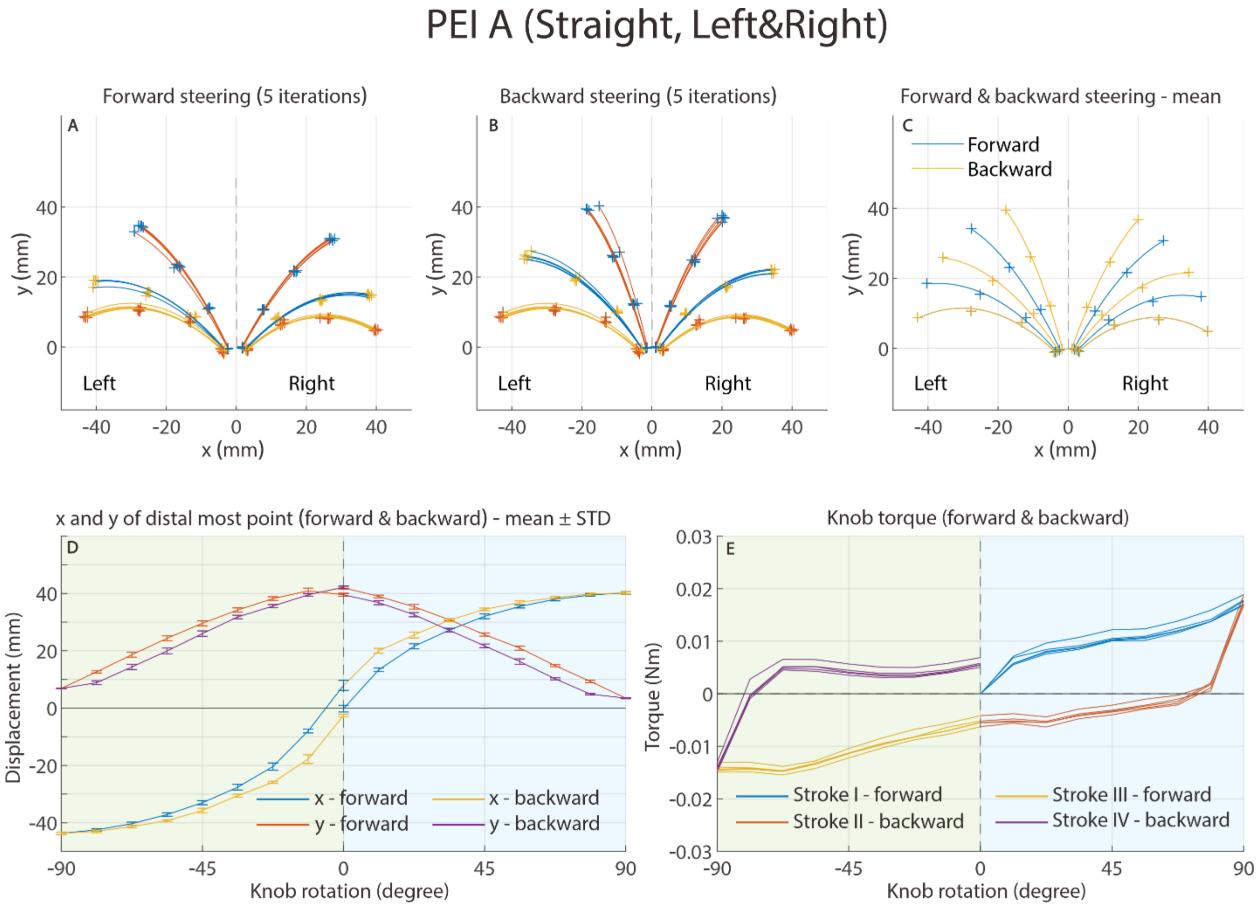


**Figure S17. The results of Experiments 4-1 show the left and right tip motion of PEI A catheter in a straight configuration.** (**A**) The geometry of the catheter for each trial when the catheter is steered forward (Stroke I and Stroke III). (**B**) The geometry of the catheter for each trial when the catheter is steered backward (Stroke II and Stroke IV). (**C**) The catheter profiles during the steering at each step (with a step length of 22.5°) are plotted by averaging the catheter positions over multiple trials with different knob angular positions. Backlash is indicated by the difference in catheter positions for the same knob angle position during forward and backward strokes. To aid visualization, only shape diagrams for knob rotations of ± 22.5°, ± 56.25°, and ± 90°are shown. (**D**) displays the average coordinates (with standard deviation) of the catheter tip positions in the x and y directions throughout the experiment. (**E**) displays the torque applied to the knob during the experimental procedure, along with its standard deviation.

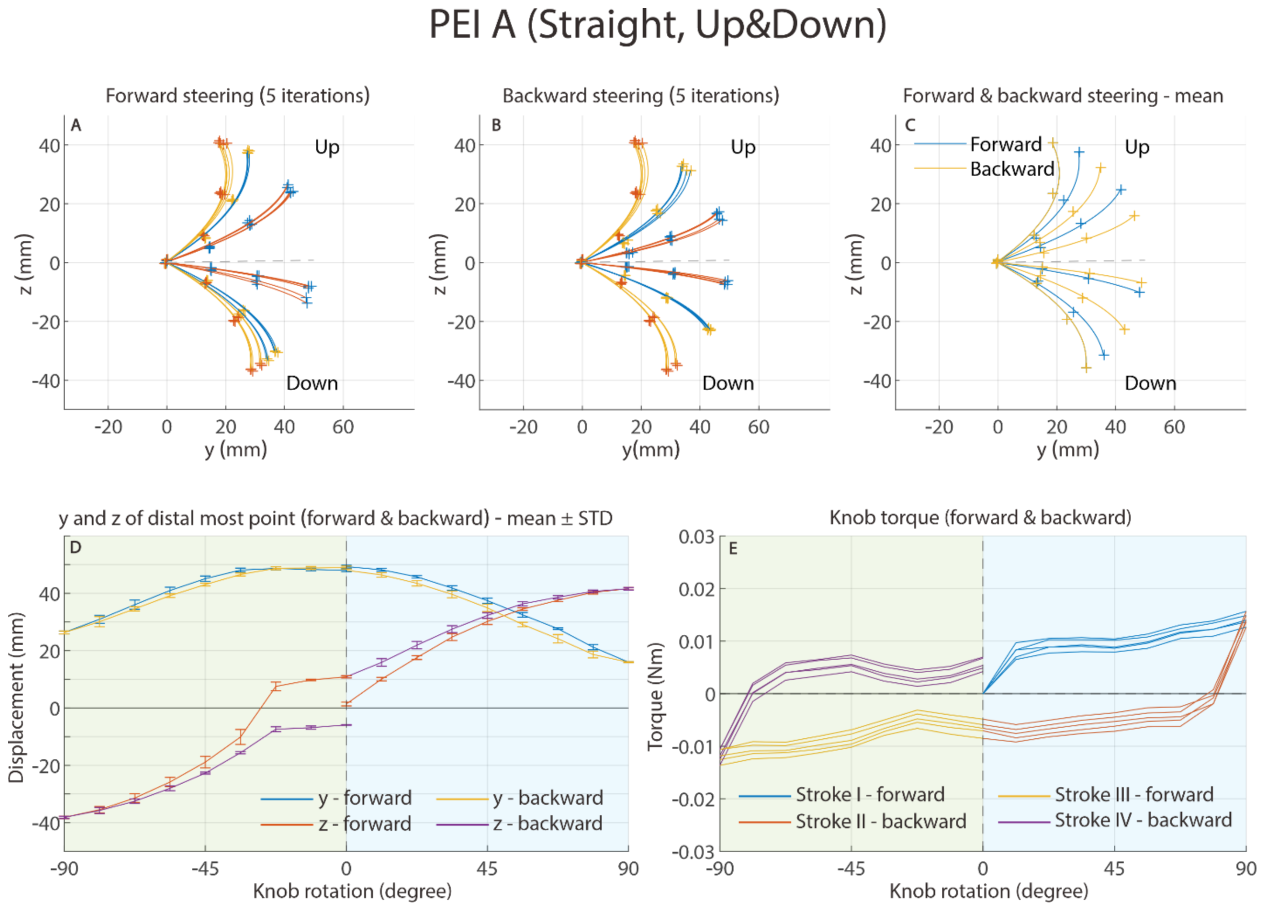


**Figure S18. The results of Experiments 4-1 show the up and down tip motion of PEI A catheter in a straight configuration.** (**A**) The geometry of the catheter for each trial when the catheter is steered forward (Stroke I and Stroke III). (**B**) The geometry of the catheter for each trial when the catheter is steered backward (Stroke II and Stroke IV). (**C**) The catheter profiles during the steering at each step (with a step length of 22.5°) are plotted by averaging the catheter positions over multiple trials with different knob angular positions. Backlash is indicated by the difference in catheter positions for the same knob angle position during forward and backward strokes. To aid visualization, only shape diagrams for knob rotations of ± 22.5°, ± 56.25°, and ± 90°are shown. (**D**) displays the average coordinates (with standard deviation) of the catheter tip positions in the x and y directions throughout the experiment. (**E**) displays the torque applied to the knob during the experimental procedure, along with its standard deviation.

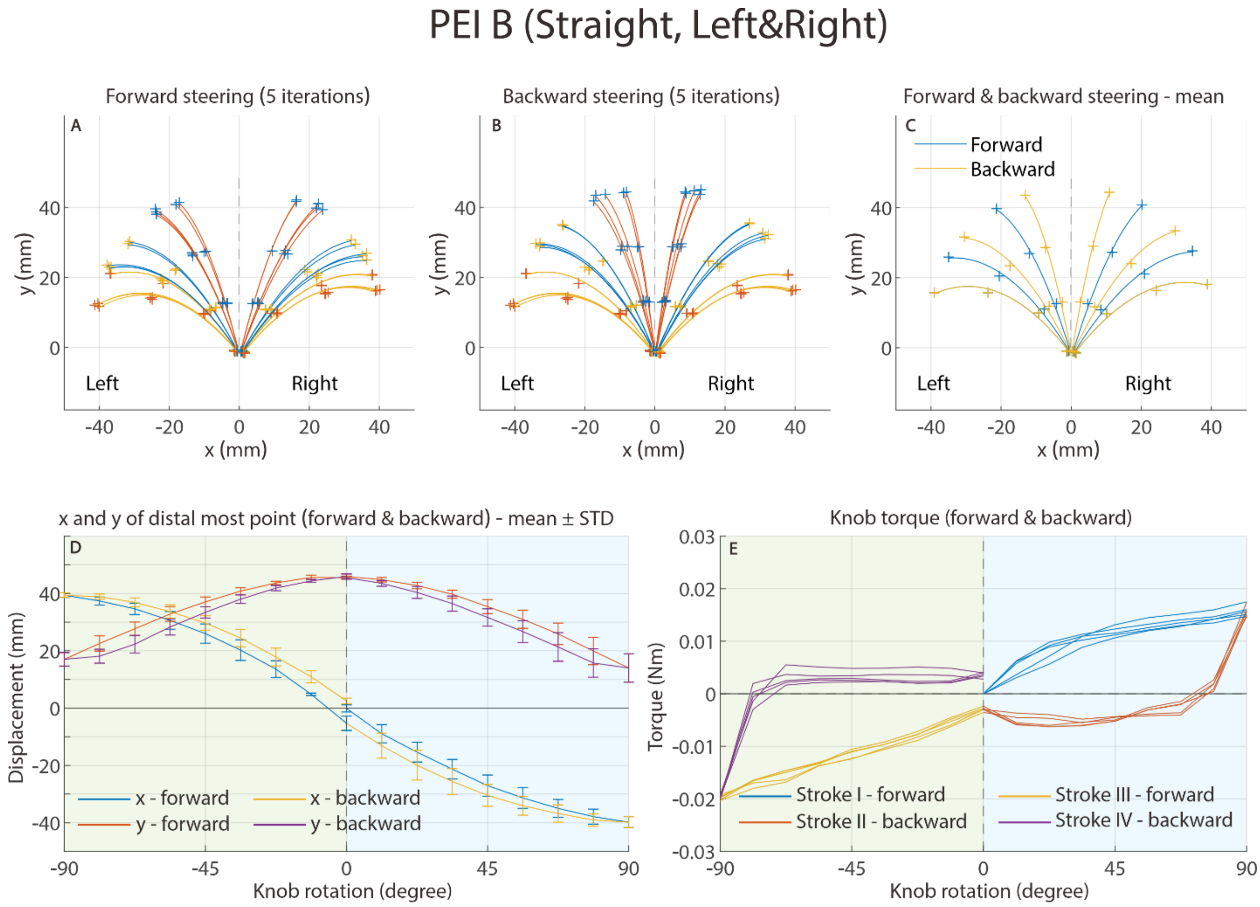


**Figure S19. The results of Experiments 4-1 show the left and right tip motion of PEI B catheter in a straight configuration.** (**A**) The geometry of the catheter for each trial when the catheter is steered forward (Stroke I and Stroke III). (**B**) The geometry of the catheter for each trial when the catheter is steered backward (Stroke II and Stroke IV). (**C**) The catheter profiles during the steering at each step (with a step length of 22.5°) are plotted by averaging the catheter positions over multiple trials with different knob angular positions. Backlash is indicated by the difference in catheter positions for the same knob angle position during forward and backward strokes. To aid visualization, only shape diagrams for knob rotations of ± 22.5°, ± 56.25°, and ± 90°are shown. (**D**) displays the average coordinates (with standard deviation) of the catheter tip positions in the x and y directions throughout the experiment. (**E**) displays the torque applied to the knob during the experimental procedure, along with its standard deviation.

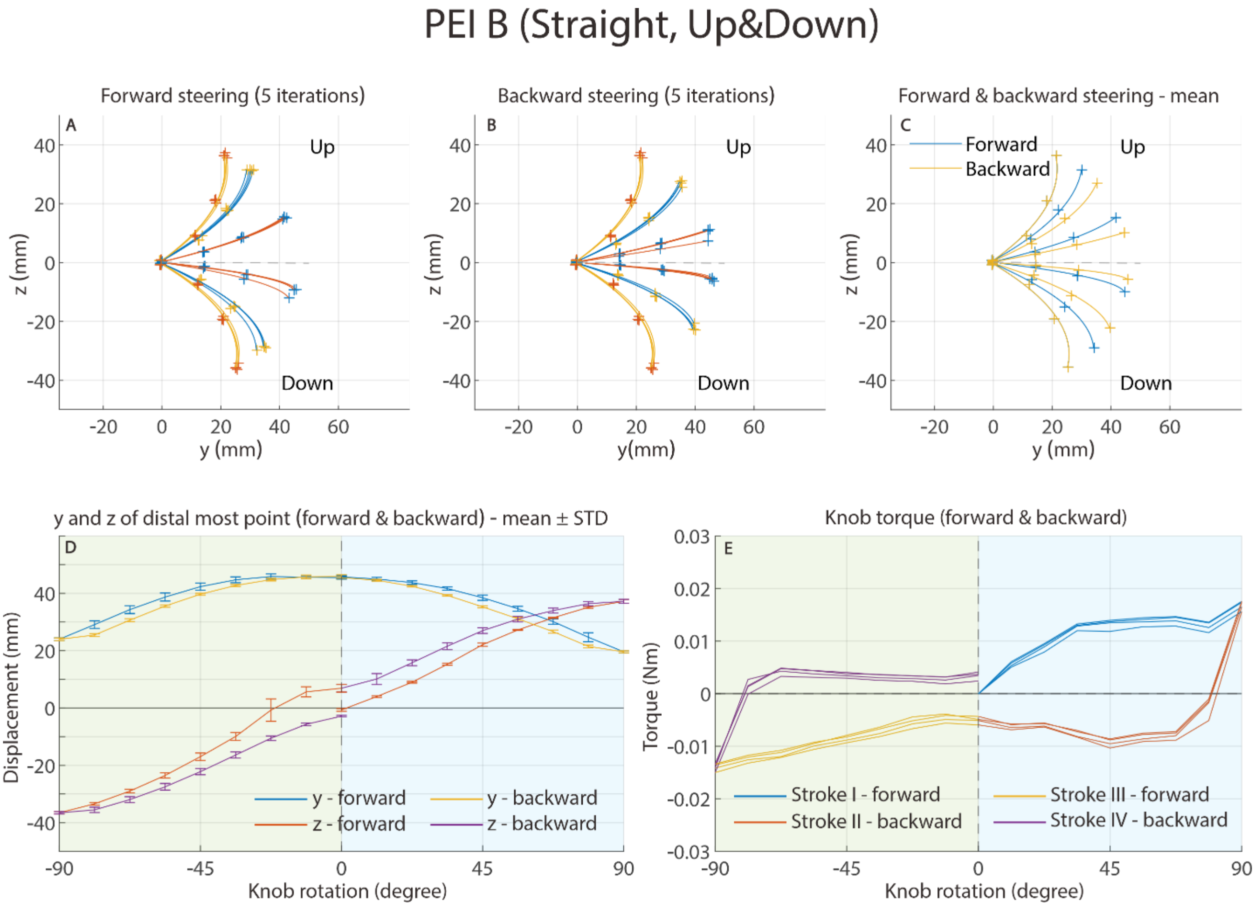


**Figure S20. The results of Experiments 4-1 show the up and down tip motion of PEI B catheter in a straight configuration.** (**A**) The geometry of the catheter for each trial when the catheter is steered forward (Stroke I and Stroke III). (**B**) The geometry of the catheter for each trial when the catheter is steered backward (Stroke II and Stroke IV). (**C**) The catheter profiles during the steering at each step (with a step length of 22.5°) are plotted by averaging the catheter positions over multiple trials with different knob angular positions. Backlash is indicated by the difference in catheter positions for the same knob angle position during forward and backward strokes. To aid visualization, only shape diagrams for knob rotations of ± 22.5°, ± 56.25°, and ± 90°are shown. (**D**) displays the average coordinates (with standard deviation) of the catheter tip positions in the x and y directions throughout the experiment. (**E**) displays the torque applied to the knob during the experimental procedure, along with its standard deviation.

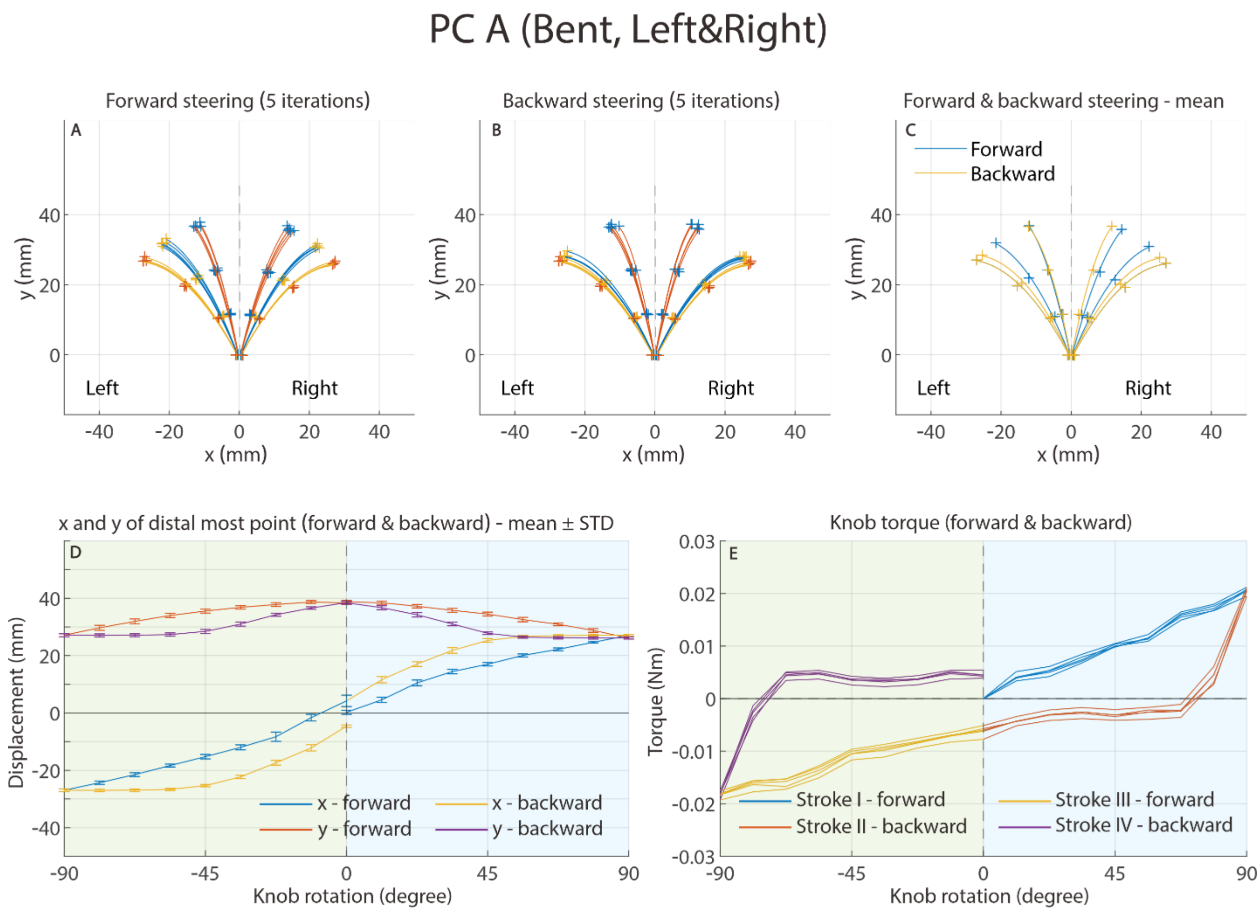


**Figure S21. The results of Experiments 4-2 show the left and right tip motion of PC A catheter in a bent configuration.** (**A**) The geometry of the catheter for each trial when the catheter is steered forward (Stroke I and Stroke III). (**B**) The geometry of the catheter for each trial when the catheter is steered backward (Stroke II and Stroke IV). (**C**) The catheter profiles during the steering at each step (with a step length of 22.5°) are plotted by averaging the catheter positions over multiple trials with different knob angular positions. Backlash is indicated by the difference in catheter positions for the same knob angle position during forward and backward strokes. To aid visualization, only shape diagrams for knob rotations of ± 22.5°, ± 56.25°, and ± 90°are shown. (**D**) displays the average coordinates (with standard deviation) of the catheter tip positions in the x and y directions throughout the experiment. (**E**) displays the torque applied to the knob during the experimental procedure, along with its standard deviation.

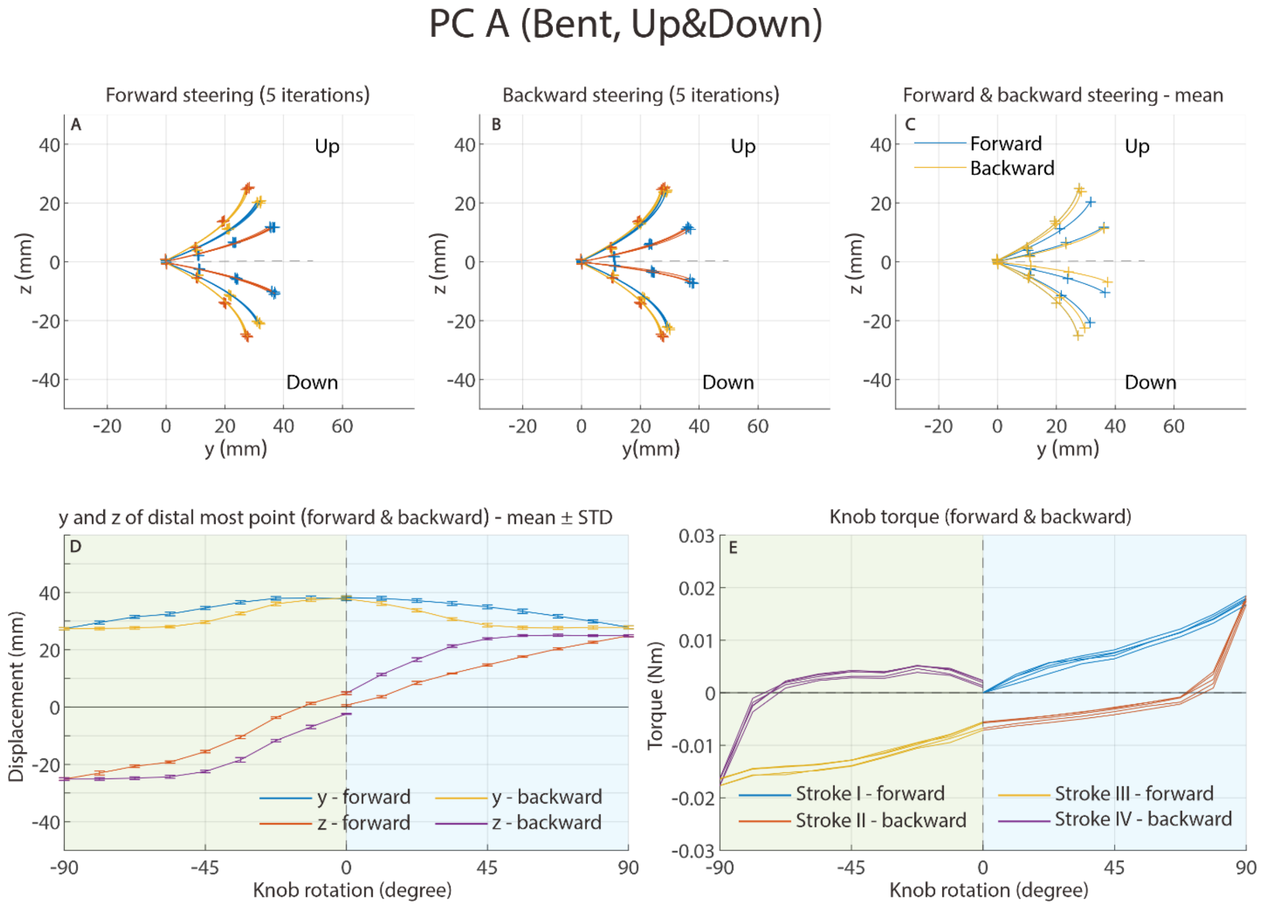


**Figure S22. The results of Experiments 4-2 show the up and down tip motion of PC A catheter in a bent configuration.** (**A**) The geometry of the catheter for each trial when the catheter is steered forward (Stroke I and Stroke III). (**B**) The geometry of the catheter for each trial when the catheter is steered backward (Stroke II and Stroke IV). (**C**) The catheter profiles during the steering at each step (with a step length of 22.5°) are plotted by averaging the catheter positions over multiple trials with different knob angular positions. Backlash is indicated by the difference in catheter positions for the same knob angle position during forward and backward strokes. To aid visualization, only shape diagrams for knob rotations of ± 22.5°, ± 56.25°, and ± 90°are shown. (**D**) displays the average coordinates (with standard deviation) of the catheter tip positions in the x and y directions throughout the experiment. (**E**) displays the torque applied to the knob during the experimental procedure, along with its standard deviation.

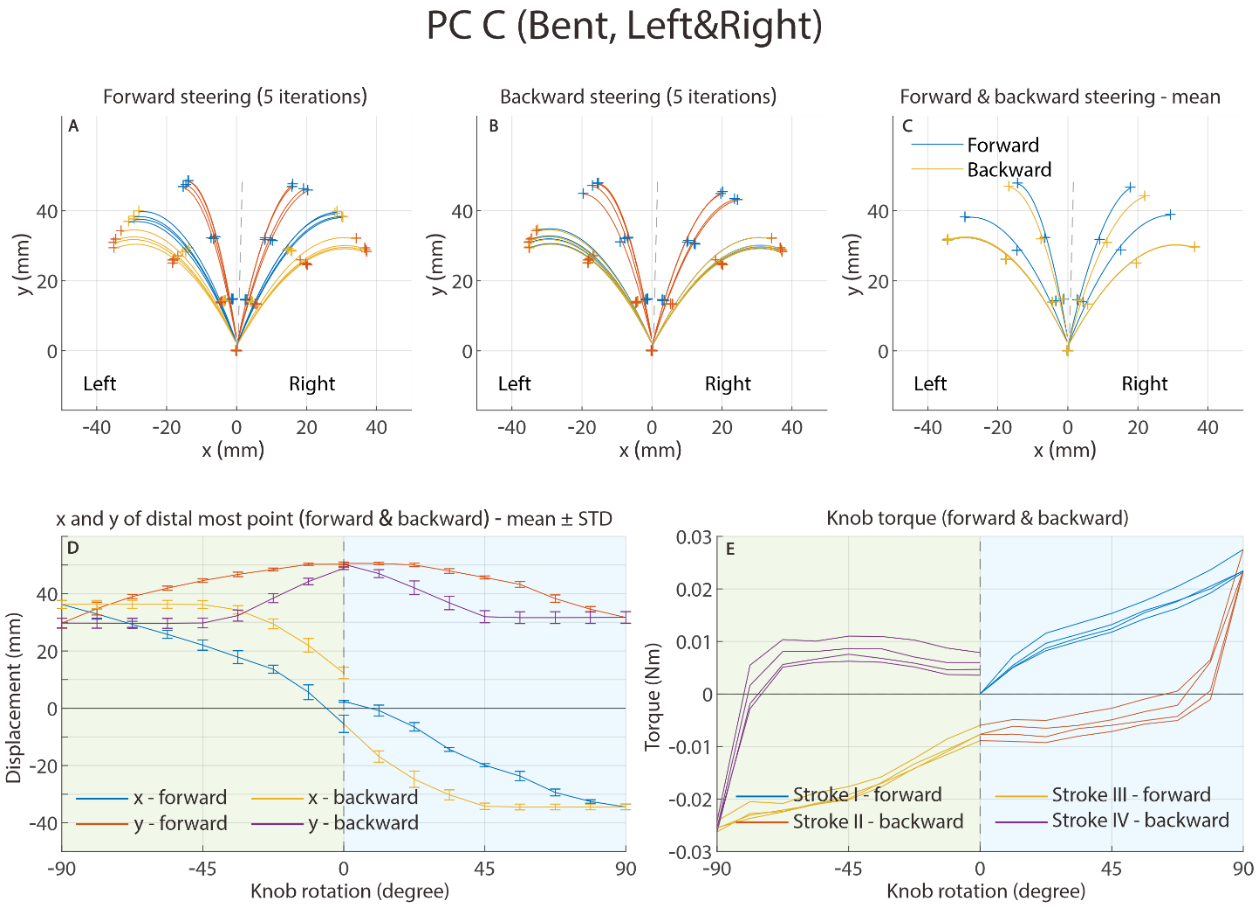


**Figure S23. The results of Experiments 4-2 show the left and right tip motion of PC C catheter in a bent configuration.** (**A**) The geometry of the catheter for each trial when the catheter is steered forward (Stroke I and Stroke III). (**B**) The geometry of the catheter for each trial when the catheter is steered backward (Stroke II and Stroke IV). (**C**) The catheter profiles during the steering at each step (with a step length of 22.5°) are plotted by averaging the catheter positions over multiple trials with different knob angular positions. Backlash is indicated by the difference in catheter positions for the same knob angle position during forward and backward strokes. To aid visualization, only shape diagrams for knob rotations of ± 22.5°, ± 56.25°, and ± 90°are shown. (**D**) displays the average coordinates (with standard deviation) of the catheter tip positions in the x and y directions throughout the experiment. (**E**) displays the torque applied to the knob during the experimental procedure, along with its standard deviation.

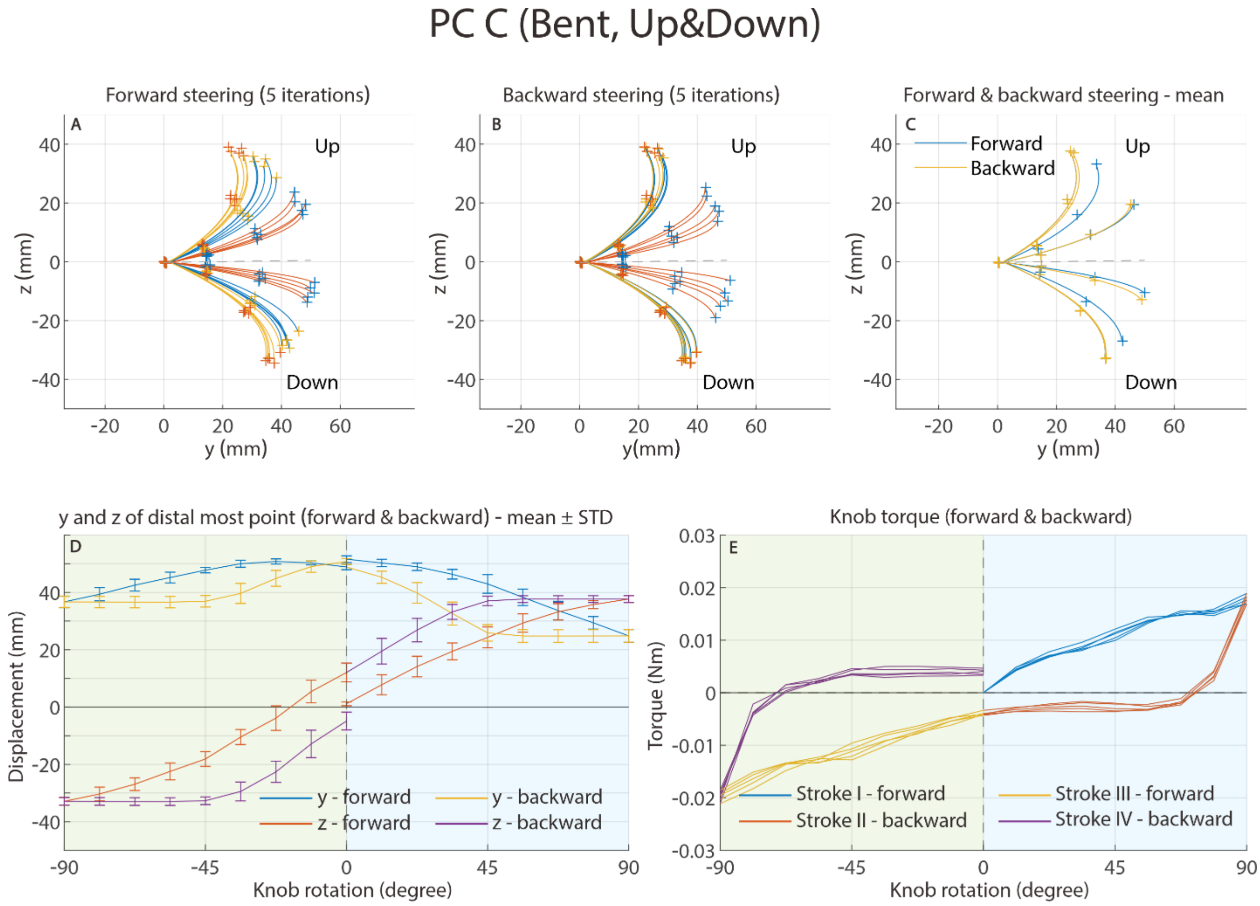


**Figure S24. The results of Experiments 4-2 show the up and down tip motion of PC C catheter in a bent configuration.** (**A**) The geometry of the catheter for each trial when the catheter is steered forward (Stroke I and Stroke III). (**B**) The geometry of the catheter for each trial when the catheter is steered backward (Stroke II and Stroke IV). (**C**) The catheter profiles during the steering at each step (with a step length of 22.5°) are plotted by averaging the catheter positions over multiple trials with different knob angular positions. Backlash is indicated by the difference in catheter positions for the same knob angle position during forward and backward strokes. To aid visualization, only shape diagrams for knob rotations of ± 22.5°, ± 56.25°, and ± 90°are shown. (**D**) displays the average coordinates (with standard deviation) of the catheter tip positions in the x and y directions throughout the experiment. (**E**) displays the torque applied to the knob during the experimental procedure, along with its standard deviation.

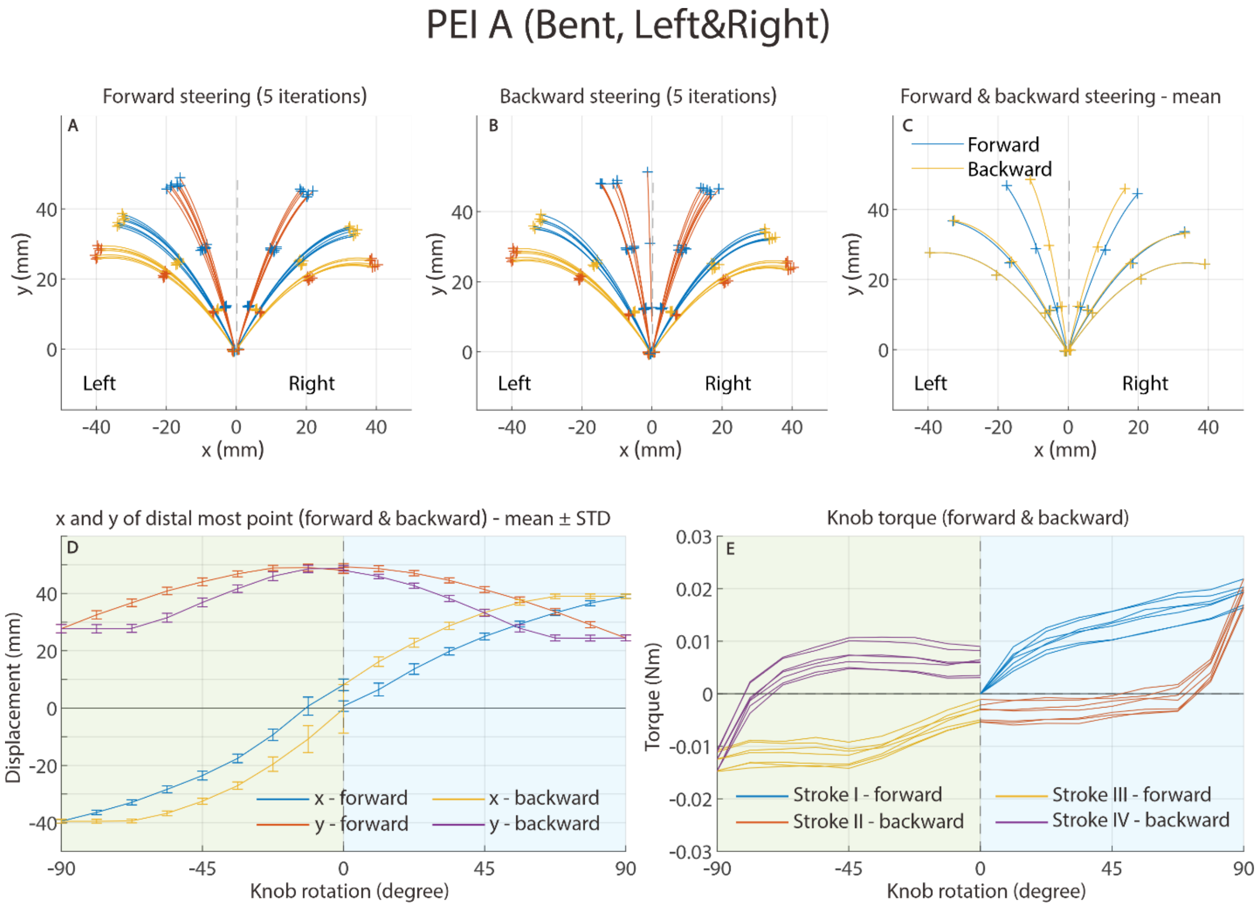


**Figure S25. The results of Experiments 4-2 show the left and right tip motion of PEI A catheter in a bent configuration.** (**A**) The geometry of the catheter for each trial when the catheter is steered forward (Stroke I and Stroke III). (**B**) The geometry of the catheter for each trial when the catheter is steered backward (Stroke II and Stroke IV). (**C**) The catheter profiles during the steering at each step (with a step length of 22.5°) are plotted by averaging the catheter positions over multiple trials with different knob angular positions. Backlash is indicated by the difference in catheter positions for the same knob angle position during forward and backward strokes. To aid visualization, only shape diagrams for knob rotations of ± 22.5°, ± 56.25°, and ± 90°are shown. (**D**) displays the average coordinates (with standard deviation) of the catheter tip positions in the x and y directions throughout the experiment. (**E**) displays the torque applied to the knob during the experimental procedure, along with its standard deviation.

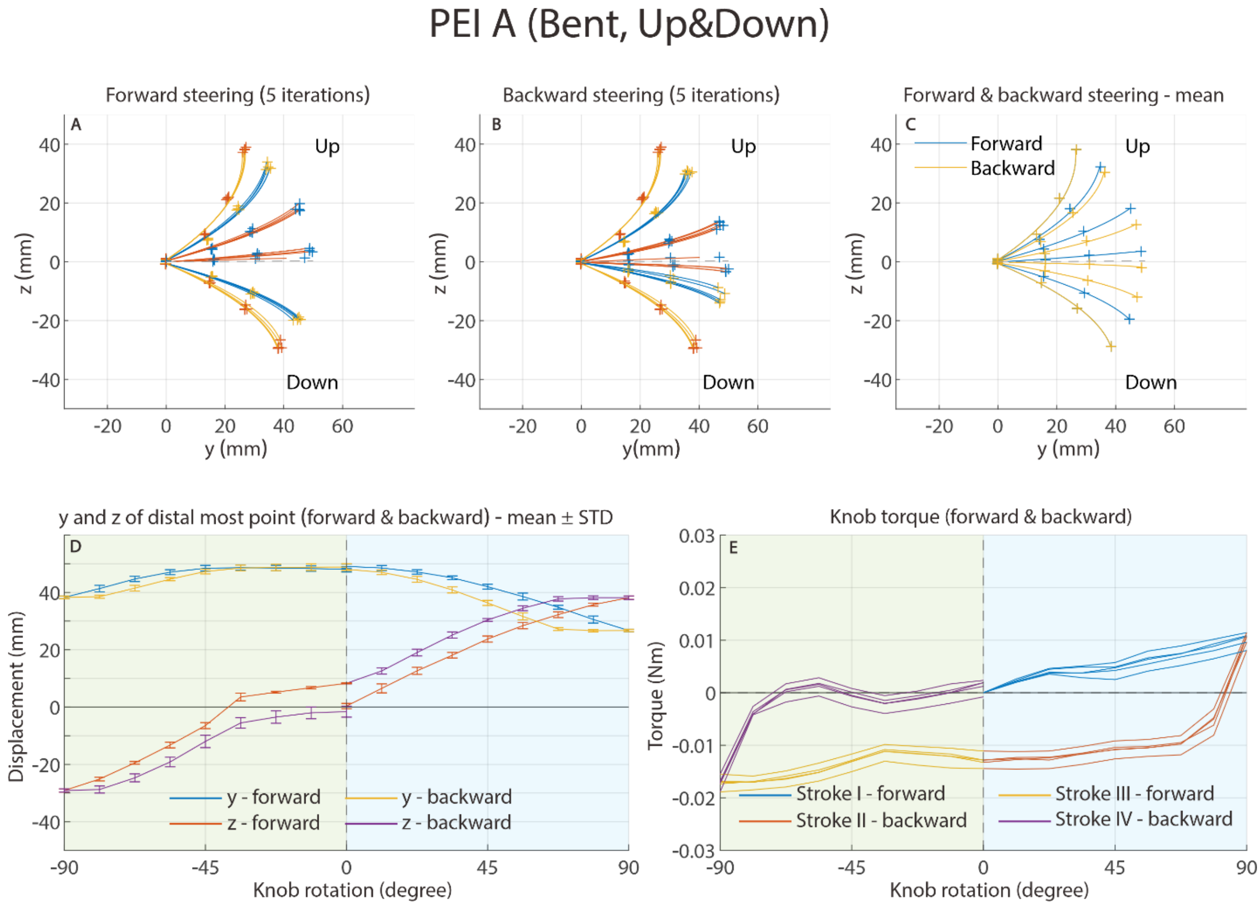


**Figure S26. The results of Experiments 4-2 show the up and down tip motion of PEI A catheter in a bent configuration.** (**A**) The geometry of the catheter for each trial when the catheter is steered forward (Stroke I and Stroke III). (**B**) The geometry of the catheter for each trial when the catheter is steered backward (Stroke II and Stroke IV). (**C**) The catheter profiles during the steering at each step (with a step length of 22.5°) are plotted by averaging the catheter positions over multiple trials with different knob angular positions. Backlash is indicated by the difference in catheter positions for the same knob angle position during forward and backward strokes. To aid visualization, only shape diagrams for knob rotations of ± 22.5°, ± 56.25°, and ± 90°are shown. (**D**) displays the average coordinates (with standard deviation) of the catheter tip positions in the x and y directions throughout the experiment. (**E**) displays the torque applied to the knob during the experimental procedure, along with its standard deviation.

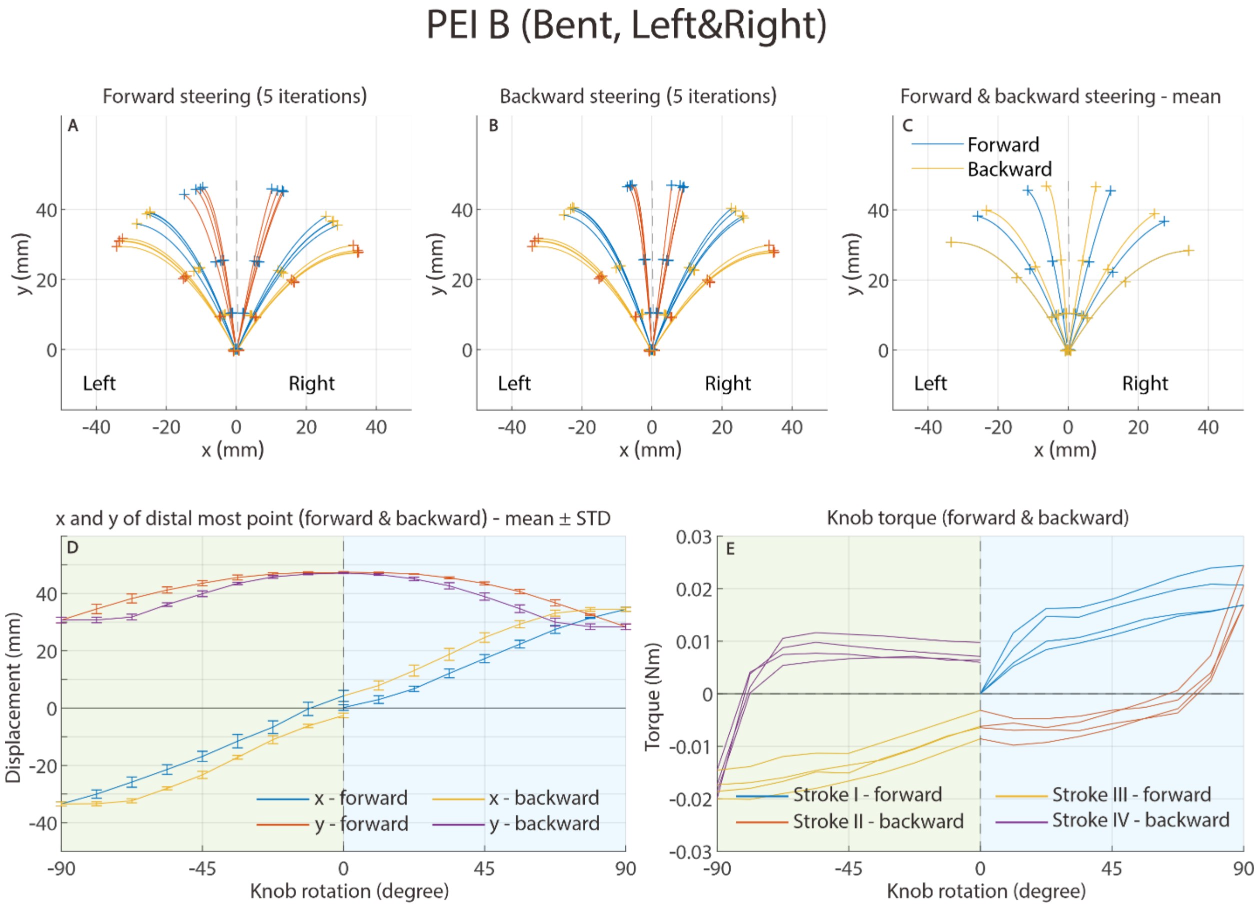


**Figure S27. The results of Experiments 4-2 show the left and right tip motion of PEI B catheter in a bent configuration.** (**A**) The geometry of the catheter for each trial when the catheter is steered forward (Stroke I and Stroke III). (**B**) The geometry of the catheter for each trial when the catheter is steered backward (Stroke II and Stroke IV). (**C**) The catheter profiles during the steering at each step (with a step length of 22.5°) are plotted by averaging the catheter positions over multiple trials with different knob angular positions. Backlash is indicated by the difference in catheter positions for the same knob angle position during forward and backward strokes. To aid visualization, only shape diagrams for knob rotations of ± 22.5°, ± 56.25°, and ± 90°are shown. (**D**) displays the average coordinates (with standard deviation) of the catheter tip positions in the x and y directions throughout the experiment. (**E**) displays the torque applied to the knob during the experimental procedure, along with its standard deviation.

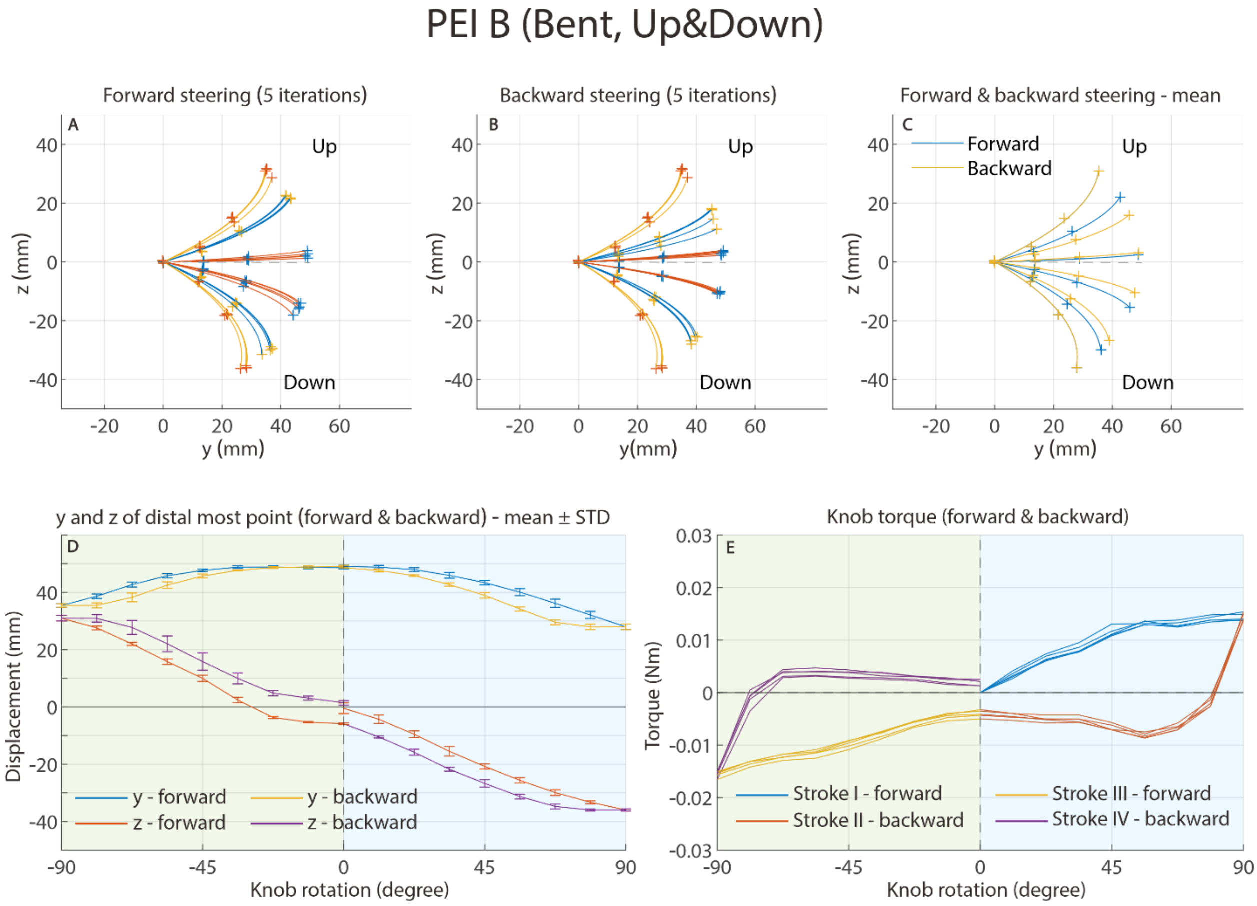


**Figure S28. The results of Experiments 4-2 show the up and down tip motion of PEI B catheter in a bent configuration.** (**A**) The geometry of the catheter for each trial when the catheter is steered forward (Stroke I and Stroke III). (**B**) The geometry of the catheter for each trial when the catheter is steered backward (Stroke II and Stroke IV). (**C**) The catheter profiles during the steering at each step (with a step length of 22.5°) are plotted by averaging the catheter positions over multiple trials with different knob angular positions. Backlash is indicated by the difference in catheter positions for the same knob angle position during forward and backward strokes. To aid visualization, only shape diagrams for knob rotations of ± 22.5°, ± 56.25°, and ± 90°are shown. (**D**) displays the average coordinates (with standard deviation) of the catheter tip positions in the x and y directions throughout the experiment. (**E**) displays the torque applied to the knob during the experimental procedure, along with its standard deviation.

**Table S1. Specifications of steerable catheters**

| Catheter No. | | PC A | PC B | PC C | PEI A | PEI B |
|---|---|---|---|---|---|---|
| Shaft | Material | PC | PC | PC | PEI | PEI |
| | Length (mm) | 990 | 850 | 870 | 945 | 750 |
| | Diameter (mm) | 2.46-2.53 | 2.35-2.41 | 2.48-2.56 | 2.7-2.88 | 2.41-2.55 |
| | Braiding | Liquid-Crystal Polymer | Kevlar | Kevlar | Kevlar | Kevlar |
| | Lumen | Straight | Helical | Helical | Straight | Straight |
| | Helix Pitch (mm) | - | 50 | 60 | - | - |
| Tip | Tip Length (mm) | 50 | 55 | 60 | 60 | 60 |
| | Tip Diameter (mm) | 2.35-2.40 | 2.25-2.30 | 2.35-2.45 | 2.6 | 2.5-2.55 |
| | Integration with Handle | Yes | No | Yes | Yes | Yes |

**Table S2. Segmented thermally drawing catheter samples**

| Sample No. | Material | Lumen | Braiding | Outer-Diameter (mm) | | Length (mm) | Experiments |
|---|---|---|---|---|---|---|---|
| | | | | min | max | | |
| 1-1 | PC | Straight | - | 2.02 | 2.09 | ~50 | 1-1; 1-3; |
| 1-2 | | | | 1.98 | 2.05 | | 1-1; 1-2; |
| 1-3 | | | | 1.87 | 1.97 | | |
| 1-4 | | | | 1.92 | 2.00 | | |
| 1-5 | | | | 1.94 | 1.97 | | |
| 1-6 | | | | 1.94 | 2.03 | ~160 | 2-1; |
| 1-7 | | | | 1.95 | 1.98 | | 3-1; |
| 2-1 | PC | Straight | Kevlar | 1.90 | 2.04 | ~50 | 1-1; 1-3; |
| 2-2 | | | | 2.13 | 2.16 | | 1-1; 1-2; |
| 2-3 | | | | 2.09 | 2.10 | | |
| 2-4 | | | | 2.13 | 2.17 | | |
| 2-5 | | | | 2.14 | 2.18 | | |
| 2-6 | | | | 2.27 | 2.40 | ~160 | 2-1; 3-1 |
| 3-1 | PC | Helical | - | 1.98 | 2.06 | ~50 | 1-1; 1-3; |
| 3-2 | | | | 1.95 | 2.00 | | 1-1; 1-2; |
| 3-3 | | | | 1.96 | 2.05 | | |
| 3-4 | | | | 1.93 | 2.03 | | |
| 3-5 | | | | 1.92 | 2.02 | | |
| 3-6 | | | | 1.96 | 2.01 | ~160 | 2-1; 3-1 |
| 3-7 | | | | 1.95 | 1.99 | | 3-1; |
| 4-1 | PC | Helical | Kevlar | 2.04 | 2.13 | ~50 | 1-1; 1-3; |
| 4-2 | | | | 2.04 | 2.12 | | 1-1; 1-2; |
| 4-3 | | | | 2.04 | 2.13 | | |
| 4-4 | | | | 2.04 | 2.10 | | |
| 4-5 | | | | 2.04 | 2.11 | | |
| 4-6 | | | | 1.83 | 2.15 | ~160 | 2-1; 3-1 |
| 5-1 | PEI | Straight | - | 2.19 | 2.27 | ~50 | 1-1; 1-3; |
| 5-2 | | | | 2.13 | 2.18 | | 1-1; 1-2; |
| 5-3 | | | | 1.92 | 1.95 | | |

| | | | | | | | |
|---|---|---|---|---|---|---|---|
| 5-4 | | | | 1.95 | 1.98 | | |
| 5-5 | | | | 1.94 | 1.95 | | |
| 5-6 | | | | 2.14 | 2.38 | ~160 | 2-1; 3-1 |
| 5-7 | | | | 2.18 | 2.27 | | 3-1; |

**Table S2 (continued). Segmented thermally drawing catheter samples**

| Sample No. | Material | Lumen | Braiding | Outer-Diameter (mm) | | Length (mm) | Experiments |
|---|---|---|---|---|---|---|---|
| | | | | min | max | | |
| 6-1 | PEI | Straight | - | 1.98 | 2.02 | ~50 | 1-1; 1-3; |
| 6-2 | | | | 1.95 | 1.98 | | 1-1; 1-2; |
| 6-3 | | | | 2.06 | 2.11 | | |
| 6-4 | | | | 2.02 | 2.06 | | |
| 6-5 | | | | 2.06 | 2.12 | | |
| 6-6 | | | | 1.76 | 1.88 | ~160 | 3-1; |
| 6-7 | | | | 1.88 | 1.93 | | 2-1; |
| 6-8 | | | | 2.18 | 2.38 | | 3-1; |

**Table S3. Segmented commercial catheter samples**

| Sample No. | Product Name | Outer-Diameter (mm) | | Length (mm) | Experiments |
|---|---|---|---|---|---|
| | | min | max | | |
| 7-1 | Magellan Guide Catheter | 1.95 | 2.02 | ~50 | 1-1; 1-3; |
| 7-2 | | 2.95 | 3.11 | | 1-1; 1-2; |
| 7-3 | | 2.84 | 3.12 | | |
| 7-4 | | 2.96 | 3.09 | | |
| 7-5 | | 2.93 | 2.96 | | |
| 7-6 | | 3.10 | 3.16 | ~160 | 2-1; 3-1 |
| 8-1 | Magellan Lead Catheter - Middle Part | 2.09 | 2.12 | ~50 | 1-1; 1-3; |
| 8-2 | | 2.03 | 2.05 | | 1-1; 1-2; |
| 8-3 | | 2.01 | 2.02 | | |
| 8-4 | | 1.97 | 2.03 | | |
| 8-5 | | 2.07 | 2.13 | | |
| 8-6 | | 2.06 | 2.13 | ~160 | 2-1; 3-1 |
| 9-1 | Magellan Lead Catheter - Proximal Part | 2.14 | 2.15 | ~50 | 1-1; 1-3; |
| 9-2 | | 2.14 | 2.17 | | 1-1; 1-2; |
| 9-3 | | 2.16 | 2.18 | | |
| 9-4 | | 2.14 | 2.16 | | |
| 9-5 | | 2.14 | 2.17 | | |
| 9-6 | | 2.15 | 2.17 | ~160 | 2-1; 3-1 |
| 10-1 | Magellan Lead Catheter - Distal Part | 1.93 | 1.94 | ~50 | 1-1; 1-3; |
| 10-2 | | 1.94 | 2.02 | | 1-1; 1-2; |
| 10-3 | | 1.91 | 1.96 | | |
| 10-4 | | 1.92 | 1.98 | | |
| 10-5 | | 1.94 | 1.98 | | |
| 10-6 | | 2.15 | 2.16 | ~160 | 2-1; 3-1 |
| 11-1 | Biosense Webster Ablation Catheter | 2.41 | 2.50 | ~50 | 1-1; 1-3; |
| 11-2 | | 2.49 | 2.51 | | 1-1; 1-2; |
| 11-3 | | 2.42 | 2.45 | | |
| 11-4 | | 2.48 | 2.54 | | |
| 11-5 | | 2.44 | 2.52 | | |

| | | | | | |
|---|---|---|---|---|---|
| 11-6 | | 2.33 | 2.47 | ~160 | 3-1; Kinked |
| 11-7 | | 2.42 | 2.45 | | 2-1; |

**Table S3 (continued). Segmented commercial catheter samples**

| Sample No. | Product Name | Outer-Diameter (mm) | | Length (mm) | Experiments |
|---|---|---|---|---|---|
| | | min | max | | |
| 13-1 | Medtronic Guide Catheter 7Fr | 2.22 | 2.33 | ~50 | 1-1; 1-3; |
| 13-2 | | 2.25 | 2.37 | | 1-1; 1-2; |
| 13-3 | | 2.25 | 2.31 | | |
| 13-4 | | 2.04 | 2.10 | | |
| 13-5 | | 2.32 | 2.46 | | |
| 13-6 | | 2.25 | 2.33 | ~160 | 3-1; Kinked |
| 13-7 | | 2.31 | 2.36 | | 3-1; Kinked |
| 13-8 | | 2.28 | 2.35 | | 3-1; Kinked |
| 13-9 | | 2.28 | 2.34 | | 2-1; |

**Table S4. Percentage decrease in *EI* of the 13 catheter shaft samples when immersed in warm water at 37.4 °C**

| Catheter Shaft Sample | ***EI* t = 0 s ($Nmm^2$)** | ***EI* t = 900 s ($Nmm^2$)** | **Drop (%)** |
|---|---|---|---|
| PC Parallel (Non-Braided) | 587.72 | 528.96 | 10.00 |
| PC Parallel (Braided) | 626.40 | 557.83 | 10. 95 |
| PC Helical (Non-Braided) | 521.05 | 478.85 | 8.10 |
| PC Helical (Braided) | 518.65 | 464.80 | 10.38 |
| PE Parallel (Non-Braided) | 767.89 | 685. 89 | 10.68 |
| PE Helical (Non-Braided) | 815.08 | 717.73 | 11.94 |
| Magellan Guide Catheter | 474.82 | 391.78 | 17.49 |
| Magellan Lead Catheter - Proximal Part | 439.68 | 376.34 | 14. 41 |
| Magellan Lead Catheter - Middle Part | 207.23 | 186.70 | 9.91 |
| Magellan Lead Catheter - Distal Part | 172.26 | 139.15 | 19.22 |
| Biosense Webster Ablation Catheter | 601 .53 | 538.45 | 10.49 |
| Medtronic Guiding Catheter 6Fr | 241.33 | 186.98 | 22.52 |
| Medtronic Guiding Catheter 7Fr | 420.31 | 325.80 | 22.49 |

**Table S5. Thermal drawing settings for materials used in the study**

| Material | Nature | $T_g$ (°C) | $T_m$ (°C) | Furnace temperature (°C) | | | Weight (g) |
|---|---|---|---|---|---|---|---|
| | | | | Top | Middle | Bottom | |
| PC | Amorphous | 145 | - | 140 | 240 | 85 | 142 |
| PEI | Amorphous | 215 | - | 220 | 340 | 95 | 142 |
| COCe | Semi-crystalline | 6 | 84 | 120 | 210 | 85 | 142 |

**Movie S1. Real-time MRI as the catheter tip is steered in all four directions.** First part: the view from the sagittal and frontal planes. Second part: the view from the transverse and sagittal planes.

**Movie S2. Real-time MRI for in-vitro abdominal phantom study with a steerable catheter.** First part: targeting the LRA. Second part: targeting the LRA with contrast agent injection. Third part: targeting the RRA with contrast agent injection. Fourth part: targeting both the LRA and RRA with contrast agent injection. Fifth part: targeting the coeliac trunk with contrast agent injection.

**Movie S3. Real-time MRI for an animal study using a steerable catheter.** The catheter is directed towards the carotid artery and crosses the aortic arch towards the aortic root.

**Movie S4. Real-time MRI for an animal study with an active tracking catheter.** The catheter crosses the aortic arch, with contrast agent for selective perfusion.
